# *MET* alterations are enriched in lung adenocarcinoma brain metastases, defining a distinct biologic subtype

**DOI:** 10.1172/JCI194708

**Published:** 2025-12-18

**Authors:** Timothy F. Burns, Sanja Dacic, Anish Chakka, Ethan Miller, Maria A. Velez, Ashwin Somasundaram, Saveri Bhattacharya, Autumn Gaither-Davis, Princey Devadassan, Jingxiao Jin, Vinod Kumar, Arjun Pennathur, Joanne Xiu, Matthew Oberley, Michael J. Glantz, Sonikpreet Aulakh, Uma R. Chandran, Riyue Bao, Curtis Tatsuoka, Laura P. Stabile

**Affiliations:** 1 Division of Hematology/Oncology, Department of Medicine, and; 2 Department of Pharmacology and Chemical Biology, University of Pittsburgh School of Medicine, Pittsburgh, Pennsylvania, USA.; 3 UPMC Hillman Cancer Center, Pittsburgh, Pennsylvania, USA.; 4 Department of Pathology,; 5 Department of Bioinformatics, and; 6 Department of Cardiothoracic Surgery, University of Pittsburgh School of Medicine, Pittsburgh, Pennsylvania, USA.; 7 Caris Life Sciences, Phoenix, Arizona, USA.; 8 Department of Neurosurgery, Penn State Health Milton S. Hershey Medical Center, Hershey, Pennsylvania, USA.; 9 Department of Medical Oncology, West Virginia University, Morgantown, West Virginia, USA.; 10 Department of Biostatistics, University of Pittsburgh School of Medicine, Pittsburgh, Pennsylvania, USA.

**Keywords:** Genetics, Oncology, Biomarkers, Lung cancer, Oncogenes

## Abstract

Non–small cell lung cancer exhibits the highest rates of brain metastases (BMs) among all solid tumors, presenting a major clinical challenge. The development of novel therapeutic strategies targeting BMs is clearly needed. We identified a significant enrichment of *MET* amplification in lung adenocarcinoma (LUAD) BMs compared with primary LUAD and extracranial metastases in oncogene driver–negative patients. Of note, *MET*-amplified BMs were responsive to MET inhibitors in vivo, including models with acquired *MET* amplification at the time of metastasis. *MET* alterations (amplifications and/or mutations) were also more frequently detected in circulating tumor DNA from patients with LUAD BMs than in those without BMs. *MET-*altered BMs also demonstrated unique genomic features compared with non–*MET-*altered BMs. Transcriptomic analyses revealed that in contrast to *MET* WT BMs, *MET*-amplified BMs exhibited a more inflamed tumor microenvironment and displayed evidence of metabolic adaptation, particularly a reliance on glycolysis in contrast to OXPHOS in *MET* WT BMs. Furthermore, *MET*-amplified BMs demonstrated evidence of epithelial-mesenchymal transition signaling, including increased expression of TWIST1. Patients with *MET*-amplified BMs had significantly shorter overall survival. These findings highlight *MET* amplification as a critical driver of LUAD BMs, emphasizing its potential as a therapeutic target.

## Abstract 2

*MET* amplification occurs more frequently in lung adenocarcinoma brain metastases than primary tumors and displays distinct molecular and transcriptomic features, supporting targeting MET in brain metastases.

## Graphical abstract

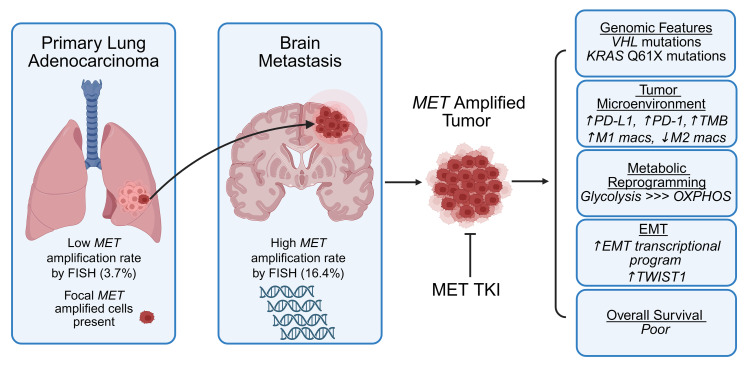

## Introduction

Lung cancer remains the leading cause of cancer-related deaths in the United States (1). Among solid tumors, non–small cell lung cancer (NSCLC) has the highest incidence of brain metastases (BMs) (2–4). Approximately 25% of patients with NSCLC present with BMs at diagnosis, and more than 40–50% eventually develop BMs during their disease course (4, 5). BMs are more common in patients with lung adenocarcinoma (LUAD) histology (5, 6), and the prognosis of patients with BMs and LUAD histology is improved compared with those with non-LUAD histologies; however, the prognosis for patients with BMs without a CNS-targetable oncogenic driver (*EGFR* mutation or *ALK*, *RET*, or *ROS1* translocation) remains poor (6–8). Advances in classifying NSCLC into molecularly defined subgroups responsive to specific therapies have shifted the treatment paradigm from standard chemotherapy to personalized targeted therapies and immunotherapy. Unfortunately, despite these advances, the brain often remains the primary site of disease progression, even in patients for whom the systemic disease is controlled by targeted therapies or immunotherapy (9–11). This underscores the urgent need for more effective treatment strategies to improve outcomes in this challenging patient population.

The HGF/MET pathway has emerged as a promising target for treatment and/or prevention of NSCLC BMs. Studies have shown increased total and phosphorylated MET expression in NSCLC BMs and high HGF levels in astrocytes (12–15). MET is a receptor tyrosine kinase that binds to HGF, activating signaling pathways that drive cell proliferation, epithelial-mesenchymal transition (EMT), motility, invasion, angiogenesis, and metastasis (16). In NSCLC, MET pathway dysregulation occurs through MET or HGF protein overexpression, *MET* amplification, or *MET* mutations (17, 18). *MET* amplification is detected in 2–4% of primary NSCLC tumors (19, 20) and is associated with poor prognosis (21, 22). It is also a well-established mechanism of acquired resistance to EGFR tyrosine kinase inhibitors (TKIs) (23, 24). Additionally, *MET* exon 14 skipping mutations (*METΔex14*) have been identified in 2–4% of NSCLC cases (20, 21, 25–28). Both *MET* amplification and *METΔex14* mutations are clinically actionable alterations in NSCLC, as dramatic responses to MET TKIs have been observed in patients with these alterations (27–35). In the GEOMETRY mono-1 study, the MET TKI capmatinib showed efficacy in extracranial lesions with a *MET* gene copy number (GCN) ≥ 10 (31). However, MET TKIs have shown overall response rates exceeding 50% with a lower cutoff (MET/CEP7 ratio ≥ 4) when assessed by FISH (32–34) or circulating tumor DNA (ctDNA) analysis (35, 36). Furthermore, MET TKI activity has been reported at an even lower cutoff (MET/CEP7 ratio ≥ 2) in the setting of *EGFR* mutant NSCLC with *MET* amplification (24). Despite these findings, the level of *MET* amplification required for MET dependency in BMs remains unclear. The MET TKIs capmatinib and tepotinib have shown preliminary evidence of activity against *METΔex14* mutant BMs, as well as in *MET-*amplified primary NSCLC (31, 35, 37, 38). Additionally, tepotinib has demonstrated efficacy in inhibiting *MET*-amplified BM growth in orthotopic preclinical models (39), and savolitinib has demonstrated activity against *MET*-amplified BMs (40). Interestingly, 2 case reports of patients with NSCLC BMs, one with a rare *MET* gene fusion found in the primary lung lesion (41) and the other with concurrent *ALK* fusion and *MET* amplification found in the BM (42), both demonstrated rapid intracranial responses to MET TKIs. It should be noted that in the second report, it is unclear whether *MET* amplification or the *ALK* fusion was the driver oncogene since both alterations are known to be sensitive to crizotinib.

Defining the molecular genotype of BMs is crucial to identifying potential therapeutic targets in patients with NSCLC BMs. However, molecular studies in BMs are limited compared with the numerous studies that have defined the molecular landscape of primary NSCLC tumors. A landmark study comparing paired primary and BMs from lung, melanoma, and breast cancers revealed that distinct targetable alterations (PI3K/AKT/mTOR, CDK, and HER2/EGFR) are enriched in BMs compared with primary lesions (43). Notably, 53% of BMs harbored clinically targetable alterations that were not detected in the paired primary tumors, though this study included only 38 lung cancer BM cases. Interestingly, in this small cohort, *MET* amplification was found in 4/34 (11.8%) of nonsquamous NSCLC BMs, with half of these cases exhibiting BM-specific *MET* amplification not detected in the primary tumor. A separate study of 73 LUAD BMs found higher amplification frequencies of *MYC*, *YAP1*, and *MMP13* compared with primary LUAD tumors in The Cancer Genome Atlas dataset (44). More recent genomic studies of NSCLC BMs have reported higher frequencies of distinct alterations, including *TP53*, *KRAS,* and *CDKN2A* mutations, in BMs compared with extracranial sites (45, 46). While one study did not assess gene amplifications (46), our previous study (45) identified a 2-fold increase in *MET* amplification in NSCLC BMs compared with primary NSCLC (4.4% vs. 2.4%). These studies utilized next-generation sequencing–based (NGS-based) platforms and GCN to determine amplification, which is less sensitive than FISH for detecting amplification, as NGS requires higher GCN cutoffs to call focal amplification and exclude aneuploidy. To date, no previous BM studies have specifically evaluated *MET* amplification using FISH.

In this study, we identified a significant enrichment in the frequency of *MET* amplification in LUAD BMs compared with both primary LUAD and liver metastases. Remarkably, these *MET* amplification events occurred in patients lacking oncogenic drivers who had not received prior targeted therapy and were not simply due to an acquired *MET* amplification at the time of resistance. Our findings reveal a distinct molecular and transcriptomic landscape of LUAD BMs, characterized by immune and metabolic adaptations as well as induction of EMT that differentiate primary LUAD from LUAD BMs, as well as *MET*-altered (amplified and/or mutated) BMs from non–*MET*-altered BMs. Furthermore, we found that patients with lung cancer with *MET-*amplified BMs have significantly worse overall survival (OS) compared with those without *MET* amplification, emphasizing the aggressive nature of these tumors. Importantly, our data suggest that a liquid biopsy approach may serve as a viable approach for detecting BM-specific *MET* alterations as these were more frequently detected in ctDNA from patients with LUAD BMs than in those without BMs. Effective treatments for patients with lung cancer BMs represent an unmet need in current oncology clinical care. Results from this study provide critical insights into the biology of MET-driven LUAD BMs and suggest that targeting *MET* amplification, along with the associated immune and metabolic pathways, could offer therapeutic opportunities for patients with LUAD BMs who lack targetable extracranial oncogenic drivers.

## Results

### Acquired MET amplification in a LUAD BM that was responsive to capmatinib.

**Figure 1:**
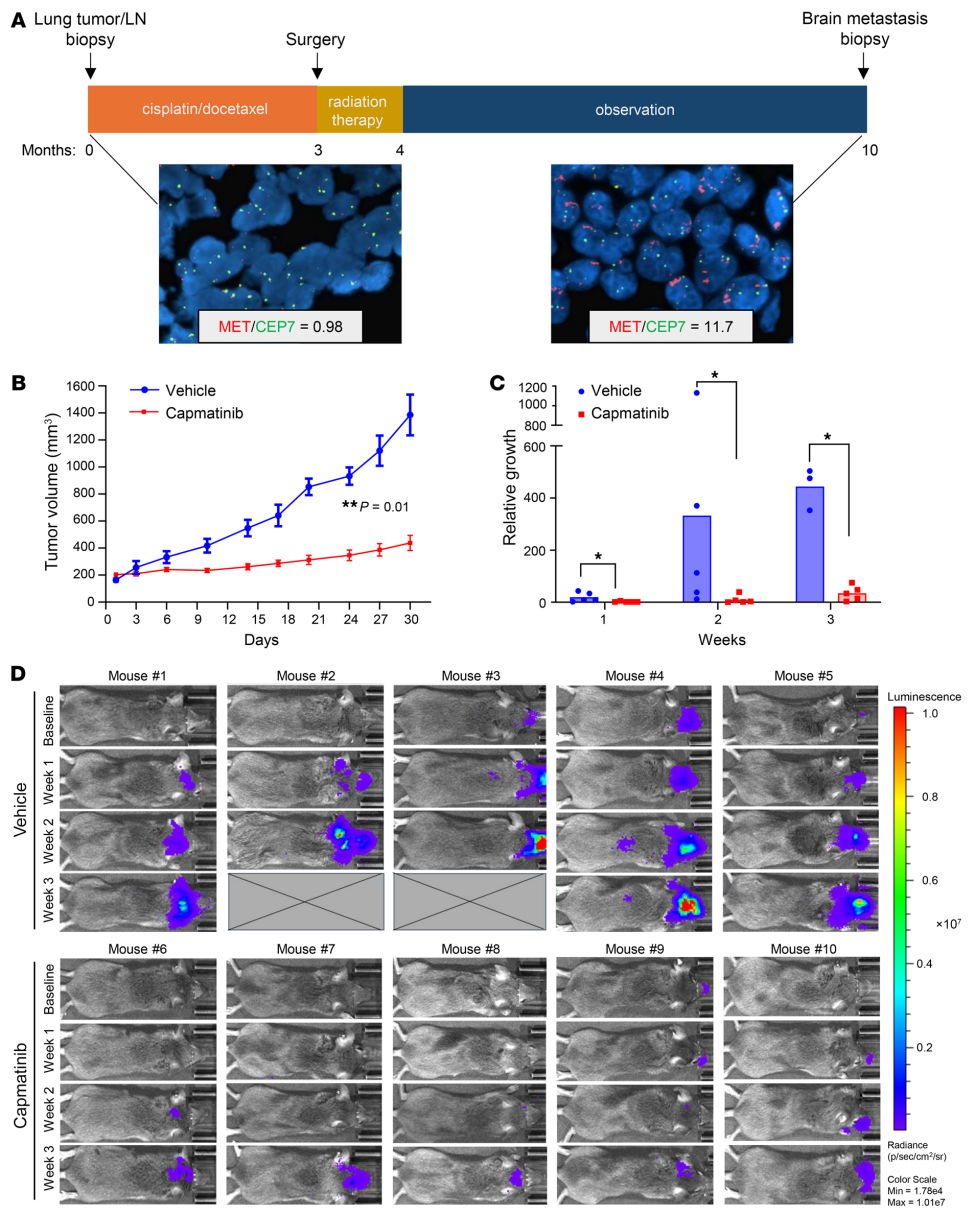
Acquired *MET* amplification in a LUAD BM that was responsive to capmatinib. (**A**) Timeline summarizing the treatment course, tumor biopsies, and *MET* amplification status. *MET* FISH images (×40 original magnification) are shown for the primary tumor biopsy at the time of diagnosis and 10 months later at the time of metastatic brain tumor biopsy. Red signals, *MET*; green signals, centromere 7 (CEP7). (**B**) A PDX model was established from patient 16-16 BM resection specimen. Mice were randomized to receive vehicle (0.25% w/v methyl cellulose) or capmatinib (5 mg/kg) by oral gavage 5 times per week for 4 weeks. Results are presented as mean tumor volume ± SEM of 6 tumors/group. Data were assessed by 2-tailed Student’s *t* test; **P = 0.01. (**C**) Luciferase-labeled H1993 LUAD cells were injected intracardially into SCID mice and monitored for metastatic spread. Mice were randomized to receive either vehicle (0.25% w/v methyl cellulose) or capmatinib (5 mg/kg) via oral gavage, administered 5 times per week for 3 weeks. Bioluminescent signal intensity in the head region was quantified relative to baseline and is presented as the mean ± SD. Statistical analysis was performed using the Mann-Whitney test; *P < 0.05. (**D**) Longitudinal bioluminescent imaging of individual mice over the course of treatment. All images were acquired and analyzed using Living Image Software (Perkin Elmer) and set to the same intensity scale for comparison. “X” represents mice that died prior to the end of the 3-week treatment period.

A patient seen in our clinics with locally advanced (stage IIIA, T3N2M0; AJCC Cancer Staging Manual, seventh edition) LUAD underwent biopsy of the primary LUAD and lymph nodes prior to treatment; no molecular testing was performed at that time. FISH for *ALK*, *MET*, *RET*, and *ROS1* were all negative for amplification or gene arrangement. The patient subsequently underwent 3 cycles of neoadjuvant cisplatin/docetaxel prior to surgery. As the patient had microscopic N2 disease after surgery, they underwent postoperative radiation followed by observation. Unfortunately, after 5 months of observation, the patient developed a BM (Figure 1A). Genotyping of the resected BM using NGS was negative for *EGFR*, *KRAS*, *BRAF*, and *PIK3CA* mutations. Programmed cell death ligand 1 (PD-L1) immunostaining was also negative. Although the primary LUAD from this patient was negative for *MET* FISH (MET/CEP7 ratio = 0.98), FISH analysis of the BM revealed *MET* amplification with a MET/CEP7 ratio of 11.7. A patient-derived xenograft (PDX) model from the brain biopsy (PDX 16-16) was generated (47), and in vivo treatment with the MET TKI capmatinib (5 mg/kg body weight) significantly reduced tumor growth by 68.4% compared with vehicle control (Figure 1B). Of note, we have previously published that the BM PDX 16-16 expressed high levels of pMET (47), and in Supplemental Figure 1 (supplemental material available online with this article; https://doi.org/10.1172/JCI194708DS1), we have demonstrated that tumors from this experiment had high levels of total MET and pMET expression and that pMET is significantly inhibited after capmatinib treatment. This case report highlights the discordance between the molecular profiles of primary LUAD and its corresponding BM and the potential of *MET* amplification as a therapeutic target in BMs.

To assess the therapeutic effects of MET inhibition in the context of brain metastasis, we utilized an intracardiac injection metastasis model using *MET*-amplified H1993 LUAD cells. Of note, this cell line acquired a *MET* amplification during metastasis to the lymph node as the cell line derived from the primary tumor (H2073) in the same patient lacked a *MET* amplification (48). Following injection, metastatic progression was monitored weekly using in vivo bioluminescence imaging, with treatment initiated upon detection of a predefined signal intensity in the head region, typically the first site of metastasis of this cell line. Imaging was performed weekly for 3 weeks. Ex vivo imaging confirmed BM presence in all mice included in the study. Across all time points, signal intensity in the head region was significantly higher in the vehicle-treated mice compared with those receiving capmatinib (Figure 1, C and D). Notably, 2 mice from the control group succumbed to BM-related complications prior to the final imaging time point. This finding demonstrates that MET inhibition significantly suppresses BM outgrowth of *MET-*amplified LUAD cells.

### MET amplification is more frequently observed in LUAD BMs compared with extracranial metastases and primary LUAD.

**Figure 2:**
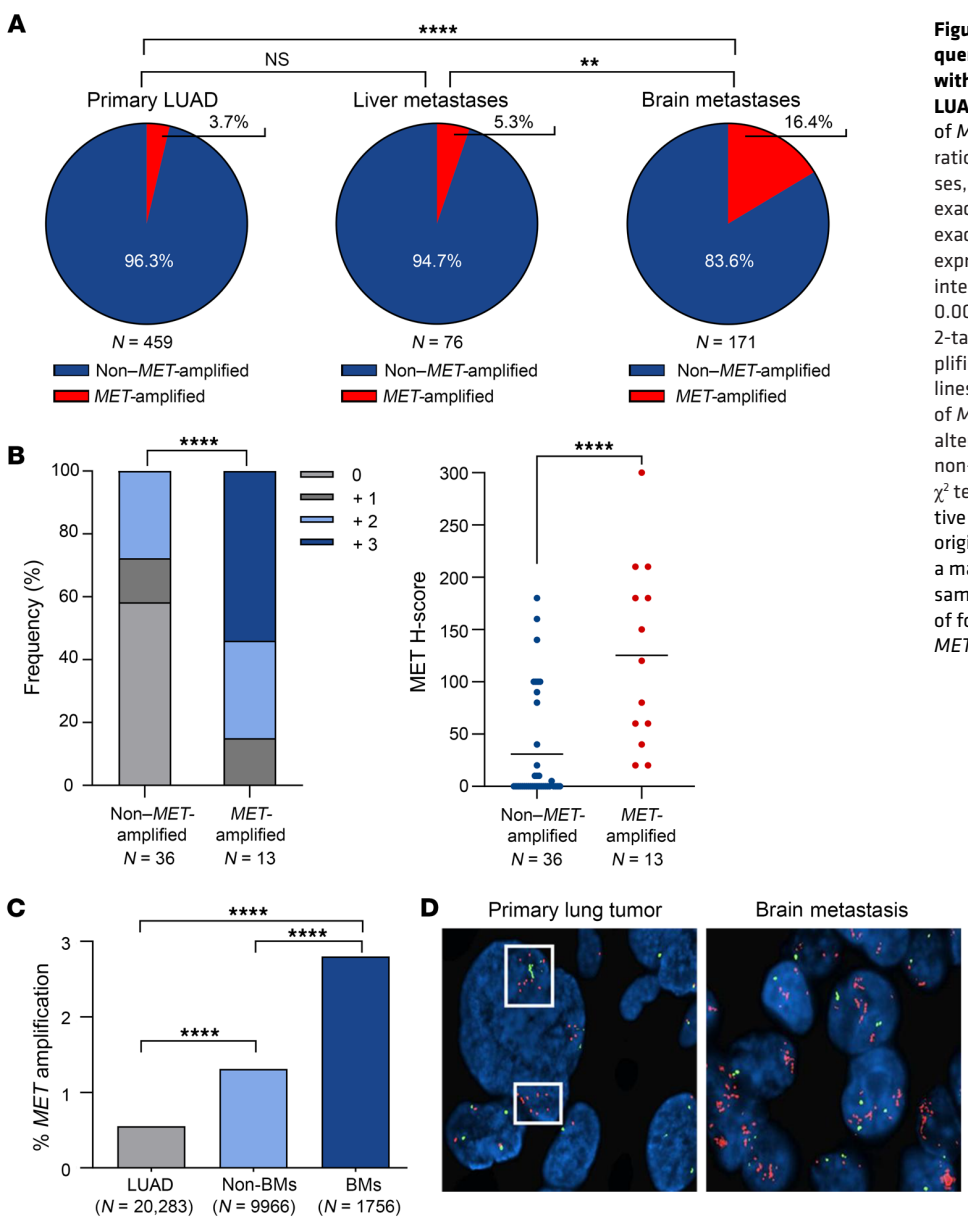
*MET* amplification is more frequently observed in LUAD BMs compared with extracranial metastases and primary LUAD. (**A**) Pie charts showing the frequency of *MET* amplification by FISH (MET/CEP7 ratio ≥ 2.0) in primary LUAD, liver metastases, and BMs in the UPMC cohort. Fisher’s exact test, 2-sided; **P < 0.01 (*P* = 0.002 exact), ****P < 0.00001. (**B**) MET protein expression by frequency of IHC staining intensity (0, +1, +2, +3; χ^2^ test; ****P < 0.0001) and MET H-score (Student’s *t* test, 2-tailed; ****P < 0.0001) in non–*MET*-amplified and *MET*-amplified BMs. Horizontal lines represent mean values. (**C**) Frequency of *MET* amplification by NGS copy number alteration (cutoff ≥ 6) in primary NSCLC, non-BMs, and BMs in the Caris cohort. χ^2^ test; ****P < 0.0001. (**D**) Representative *MET* FISH images (captured at ×40 original magnification and enlarged) from a matched primary LUAD and BM from the same patient. White boxes represent areas of focal *MET* amplification. Red signals, *MET*; green signals, CEP7.

To understand if this molecular divergence observed between primary LUAD and BMs was a frequent event, we evaluated a large cohort of patients to assess the frequency and clinical impact of *MET* amplification in metastatic sites. Previous studies assessing *MET* amplification in lung cancer BMs primarily used NGS-based platforms, which are less reliable than FISH and require higher GCN cutoffs to detect amplification and exclude aneuploidy. We therefore evaluated 459 primary LUAD, 171 LUAD BMs, and 76 liver metastases for *MET* amplification using FISH (MET/CEP7 ratio ≥ 2) (Table 1). We demonstrated that *MET* amplification was significantly enriched in LUAD BMs (16.4%) compared with primary LUAD (3.7%; *P* < 0.0001) or liver metastases (5.3%; *P* = 0.022), suggesting *MET* amplification may be a frequent and potentially targetable alteration in LUAD BMs (Figure 2A). We performed MET IHC in 49/171 (29%) BMs, including 36 non–*MET*-amplified and 11 *MET*-amplified BM cases. Of note, this subset of patients appears to be representative of the larger cohort in terms of patient characteristics (Supplemental Table 1). We observed a statistically significant increase in MET expression in the *MET-*amplified group, as assessed by both staining intensity and H-score (*P* < 0.0001) (Figure 2B). We found that high *MET* amplification (MET/CEP7 ratio ≥ 4) was present in 6.5% of BMs versus 1.3% of primary LUADs. In addition, in a subset of 31 paired primary LUAD and BM samples, *MET* amplification was present in 3/31 (10%) BMs, while none of the matched primary tumors were amplified. Remarkably, the presence of a targetable oncogenic driver was an infrequent event in these patients, and there were no cases in which a prior targeted therapy had been received. Among the 5 BM cases with an *EGFR* mutation and 1 with *ALK* rearrangement, none had a *MET* amplification, while 1 *EGFR* mutant case had a *MET* non–exon 14 skipping mutation. The demographics and clinical characteristics of the BM cohort, stratified by *MET* amplification status, are summarized in Table 2. Patients with *MET*-amplified BMs were more likely to be female, and the overwhelming majority of these patients were current/former smokers compared with those with non–*MET*-amplified BMs. There was no significant difference in the timing (synchronous vs. metachronous) of BMs between patients with and without *MET* amplification. We validated these findings using an NGS dataset from Caris Life Sciences with over 30,000 patients, demonstrating that *MET* amplification is 5 times more frequent in BMs compared with primary LUAD (*P* < 0.0001) and 2.2 times more frequent than in extracranial sites (non-BMs) (*P* < 0.0001; *MET* copy number ≥ 6) (Figure 2C).

We then asked if *MET* amplification was a rare preexisting event in the primary tumor that was subsequently enriched in the resulting BMs or whether it was truly a de novo event. Interestingly, we found examples in our matched primary LUAD and BM sets where rare cells from the primary lung tumor had focal clusters of amplified cells (Figure 2D). Rare *MET*-amplified clones likely preexist in the primary tumor, as focal *MET* amplification was observed in primary LUADs of patients with *MET*-amplified BMs (Figure 2D, white boxes). Since we found evidence of focal *MET* amplification in the primary tumor, we sought to determine whether this finding predicted the development of BMs. To assess this, we identified a cohort of NSCLC patients with and without focal *MET* amplification in the primary lung tumor (Supplemental Table 2). A retrospective chart review was conducted to determine the timing of metastasis and if patients developed BMs. BMs were confirmed based on imaging findings suggestive of BMs on a CT or MRI scan of the brain, a radiology report indicating BMs, or a brain biopsy confirming metastatic spread to the brain. Among the 85 patients with focal *MET* amplification, 28 (33%) developed BMs, compared with 49 (37%) of the 131 patients without focal *MET* amplification in our cohort. There was no statistically significant difference in the frequency of BMs between the 2 groups (*P* = 0.5). When evaluating the timing of metastatic spread, 29 patients (59%) in the non–*MET*-amplified group had synchronous metastases (occurred within 2 months of diagnosis), while 20 (41%) had metachronous metastases (occurred after 2 months of diagnosis). Among the patients with focal *MET* amplification, 17 (61%) had synchronous metastases and 11 (39%) had metachronous metastases. There was no significant difference in the timing of BMs between the 2 groups (*P* = 0.9). These results show that focal *MET* amplification was not a predictor of BMs nor did it influence the timing of the development of BMs in patients with NSCLC.

### MET alterations detected in ctDNA are found more often in patients with BMs.

**Figure 3:**
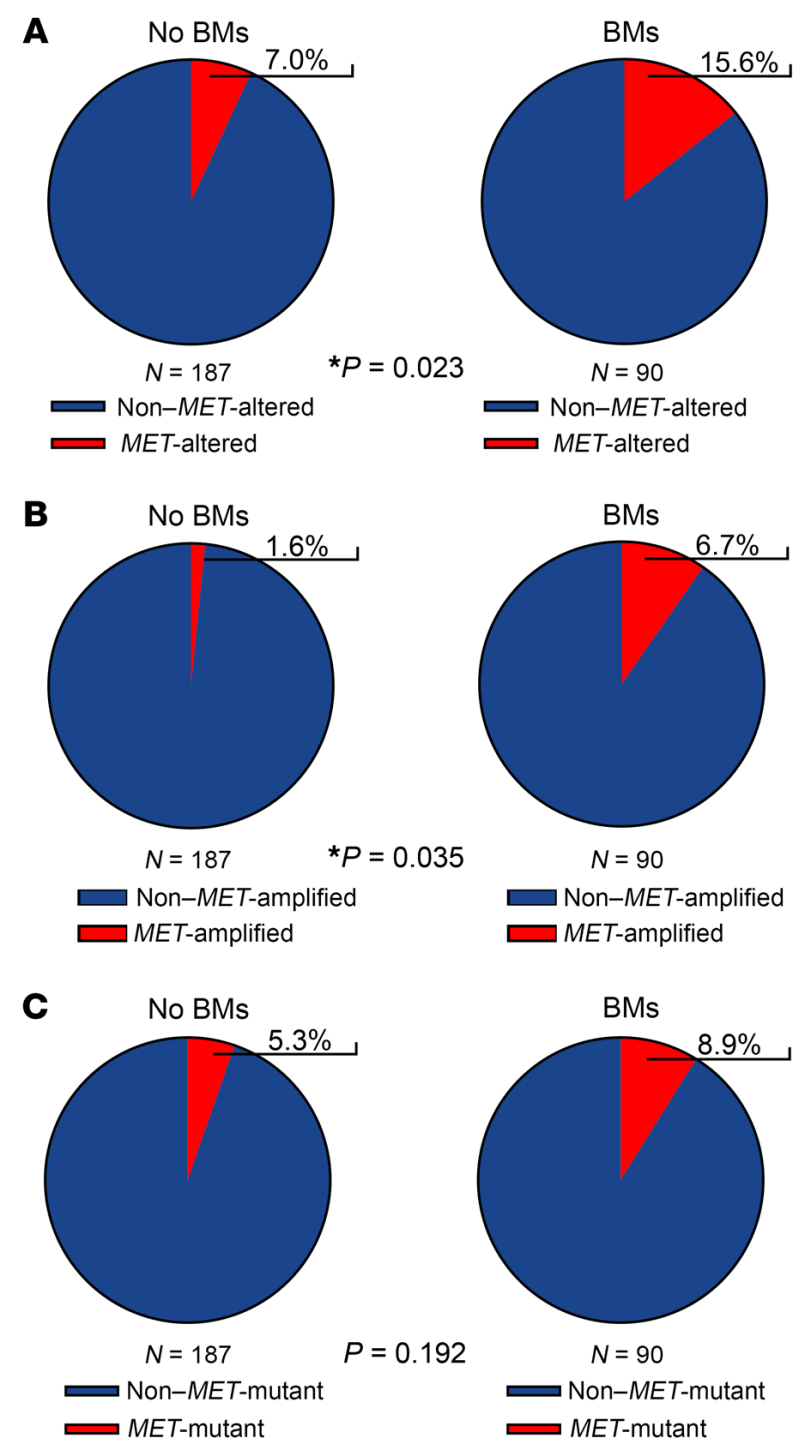
*MET* alterations detected in ctDNA are found more often in patients with BMs. (**A**) Percentage of ctDNA-positive *MET* alterations (amplifications and mutations combined) in patients with (*N* = 90) and without (*N* = 187) BMs, as detected with the Guardant360 CDx assay. (**B**) Percentage of ctDNA-positive *MET* amplifications in patients with (*N* = 90) and without (*N* = 187) BMs, as detected with the Guardant360 CDx assay. (**C**) Percentage of *MET* mutations in patients with (*N* = 90) and without (*N* =187) BMs. Fisher’s exact test, 1-sided; *P* values are shown for each comparison.

There is an unmet clinical need for noninvasive methods to detect *MET* alterations to identify patients with BMs who will benefit from MET TKIs. Although the ability of blood-based ctDNA assays to detect alterations present in BMs is diminished (49–51), we hypothesized that *MET* alterations would be more common in patients with BMs that had undergone ctDNA testing. We therefore examined a cohort of patients with metastatic NSCLC (*N* = 277) who underwent standard-of-care ctDNA testing at our institution with the Guardant360 platform to evaluate the presence of *MET* alterations in association with BMs. We observed that *MET* alterations detected by ctDNA were significantly more frequent in patients with BMs (15.6%) compared with patients without (7%) (*P* = 0.023) (Figure 3A). This appears to be driven primarily by the increased frequency of *MET* amplifications detected in patients with BMs (6.7%) compared with those without BMs (1.6%) (*P* = 0.035) (Figure 3B). While *MET* mutations were also more frequent in patients with BMs (8.9%) compared with those without BMs (5.3%), this difference was not statistically significant (Figure 3C). These findings suggest a potential role of ctDNA as a noninvasive method for detecting *MET* alterations, particularly amplifications, which may identify patients with BMs who are more likely to respond to MET TKIs.

### LUAD BMs have a distinct mutational profile compared with primary LUAD tumors.

**Figure 4:**
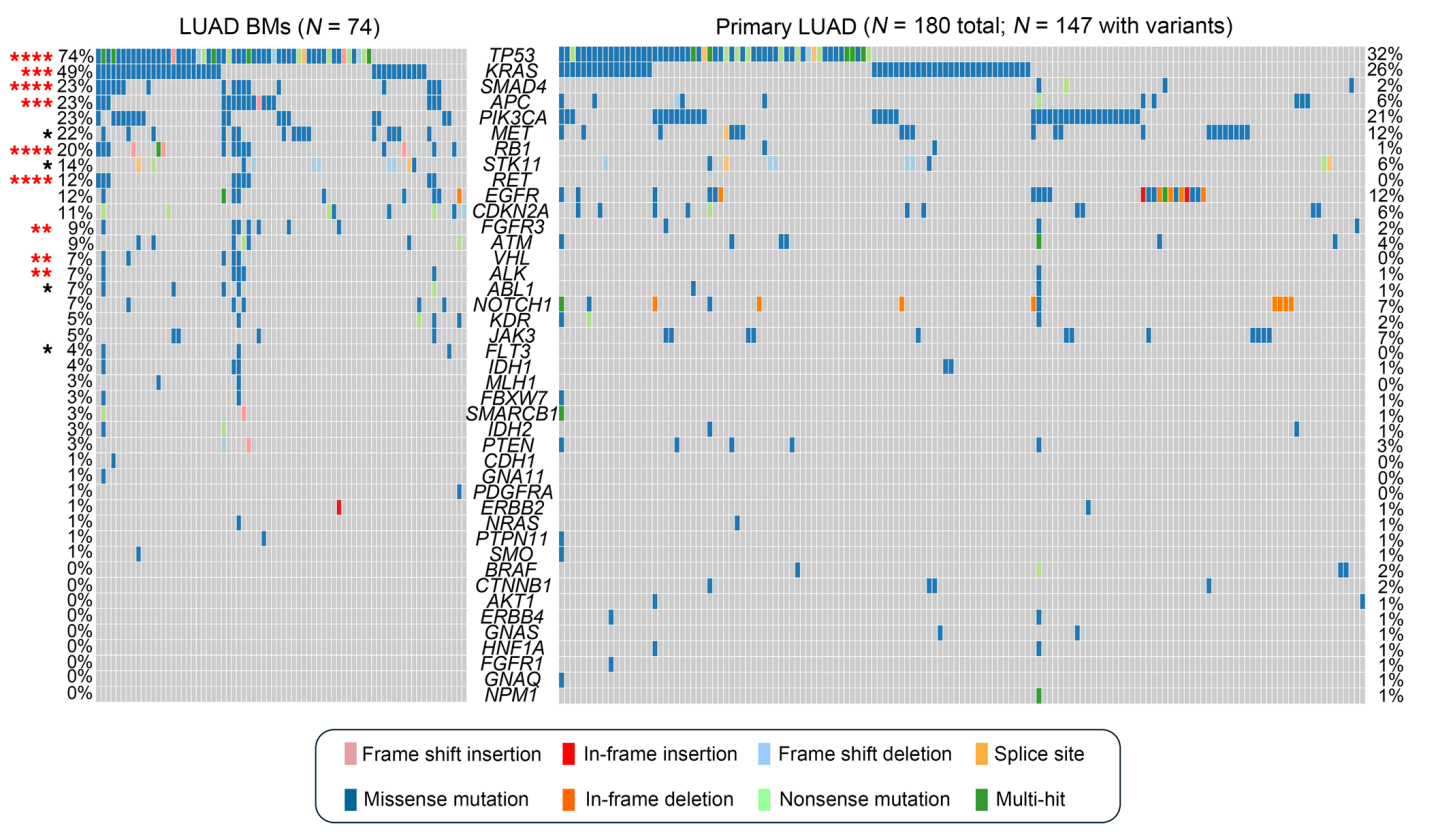
LUAD BMs have a distinct mutational profile compared with primary LUAD tumors. OncoPlot of the distribution of mutations for patients with LUAD BMs (*N* = 74) compared with those with primary LUAD tumors (*N* = 180 total; *N* = 147 with variants detected). Frequency of mutations is listed for each gene in order of the highest to lowest frequency in LUAD BMs. The mutation types are color-coded and annotated in the key. Variants annotated as “Multi-hit” are genes that are mutated more than once in the same sample. Fisher’s exact test, 2-sided; *P ≤ 0.05, **P ≤ 0.01, ***P ≤ 0.001, ****P ≤ 0.0001. Red asterisks indicate significance after FDR adjustment. A *q* value < 0.05 was considered significant.

We next performed targeted NGS to compare other alterations, including *MET* mutations, in 180 primary LUAD cases and 74 LUAD BM cases (Table 3). We found that mutations in *TP53*, *KRAS*, *SMAD4*, *APC*, *MET*, *RB1*, *STK11*, *RET*, *FGFR3*, *VHL*, *ALK*, *ABL1*, and *FLT3* were significantly more prevalent in LUAD BMs compared with primary LUAD (Figure 4). Interestingly, several of these alterations that were rare (*TP53*, *KRAS*, *MET*, *STK11*, *RET*, *FGFR3*, *VHL*, *ALK*, *ABL1*, and *FLT3*; 0–6%) or entirely absent (*SMAD4*, *APC*, and *RB1*) in primary LUAD samples were frequently observed (>20%) in LUAD BM samples. Complete lists of variants found in primary LUAD and LUAD BM cases are provided in Supplemental Tables 3 and 4, respectively. In addition, these differences were driven by specific variants that differ between these groups (Supplemental Figure 2A and Supplemental Figure 3). For example, 2 DNA-binding domain mutations in *TP53^R158L^* and *TP53^V157F^* were significantly increased in LUAD BMs compared with primary LUAD (Supplemental Figure 3A). Remarkably, the relatively rare *KRAS^Q61X^* point mutations were significantly enriched in LUAD BMs compared with primary LUAD (Supplemental Figure 3D). Of note, the frequency of *MET* mutations was significantly increased in LUAD BMs (22%) compared with primary LUAD (12%) (*P* = 0.046). Furthermore, the *MET* mutations found were predominantly non-*METΔex14*, including some mutations with unclear oncogenic potential (*MET^A179T^*, *MET^N375S^*, and *MET^T1010I^*) (Supplemental Figure 2A). We next looked at our Caris cohort, which did not include these *MET* variants. There was a significantly increased number of *MET* mutations, the majority of which were *METΔex14*, in the primary lung compared with extracranial metastatic sites (non-BMs) or BMs (Supplemental Figure 4A and Supplemental Table 5). Notably, we did not detect any *METΔex14* in our BM cohort. Interestingly, we did find a statistically higher tumor mutational burden in BMs (median 11 mut/Mb) compared with extracranial metastases (median 8 mut/Mb, *P* < 0.0001) or lung (median 7 mut/Mb, *P* < 0.0001) in the Caris cohort (Supplemental Figure 4B).

### MET-altered BMs are genomically distinct from non–MET-altered BMs.

**Figure 5:**
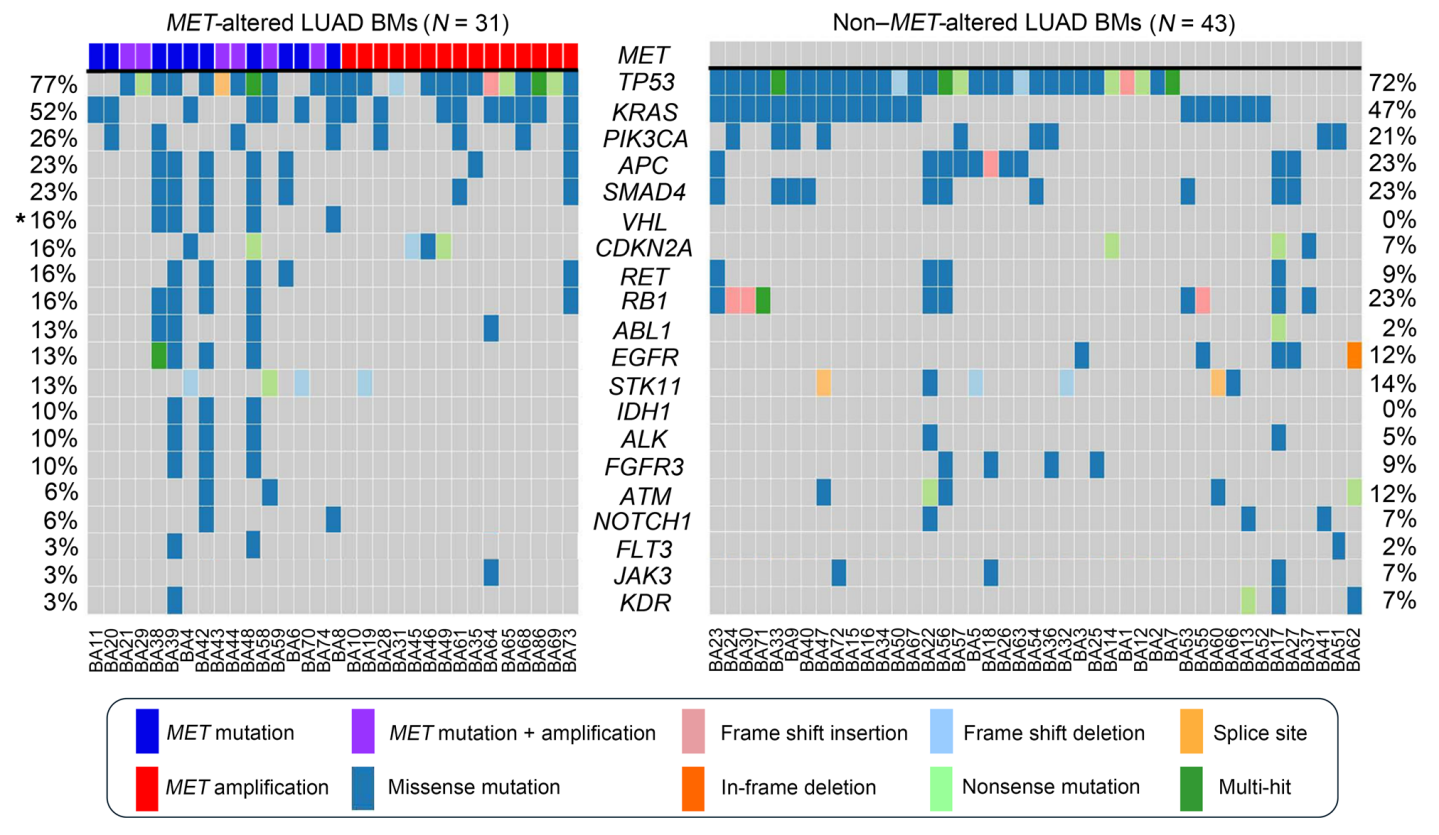
*MET-*altered BMs are genomically distinct from non–*MET-*altered BMs. OncoPlot of the distribution of mutations for patients with *MET* altered LUAD BMs (*N* = 31) compared with those with non–*MET*-altered LUAD BMs (*N* = 43). Frequency of mutations is listed for each gene in order of highest to lowest. The mutation types are color-coded and annotated in the key. Variants annotated as “Multi-Hit” are genes that are mutated more than once in the same sample. Fisher’s exact test, 1-sided; *P ≤ 0.05.

We next compared *MET*-altered (mutations and amplifications) LUAD BMs (*N* = 31) to non–*MET*-altered BMs (*N* = 43). *VHL* mutations were the only alterations that were significantly enriched in *MET*-altered BMs (16% vs. 0%, *P* = 0.01), with all identified VHL mutations co-occurring with *MET* mutations (Figure 5). Other genes that were more frequently mutated in *MET*-altered LUAD BMs but did not reach statistical significance included *CDKN2A* (16% vs. 7%; *P* = 0.19), *RET* (16% vs. 9%; *P* = 0.29), *ABL1* (13% vs. 2%; *P* = 0.09), *IDH1* (10% vs. 0%; *P* = 0.07), and *ALK* (10% vs. 5%; *P* = 0.35). Conversely, genes that were less frequently mutated in *MET*-altered cases included *ATM*, *JAK3,* and *KDR*. Of note, *KRAS^Q61X^* variants were significantly more common in *MET-*altered BMs compared with non–*MET*-altered BMs (16% vs. 2%, *P* = 0.04) (Supplemental Figure 5E). Interestingly, while genes such as *ALK*, *APC*, *FGFR3*, *IDH1*, *RB1*, and *SMAD4* were not significantly different between *MET*-altered and non–*MET*-altered BMs, they were enriched in cases with *MET* mutations compared with those with *MET* amplifications. Notably, *VHL*, *ALK*, *IDH1*, and *FGFR2* alterations were completely absent in the *MET-*amplified samples. *MET-*amplified BMs were associated with a significantly lower variant number compared with *MET* mutant BMs in our cohort (2.57 median variants per BM vs. 13.8 median variants per BM, *P* = 0.0006). The gene variants that exhibited significant differences are shown in Supplemental Figure 5. The complete list of variants for all non–*MET*-altered and *MET*-altered LUAD BM cases is shown in Supplemental Tables 6 and 7. These data suggest that *MET*-altered BMs, especially *MET*-amplified BMs, represent a molecularly and biologically distinct subset of BMs.

### BMs have distinct transcriptomic profiles of altered immune and metabolic signatures compared with primary LUAD.

**Figure 6:**
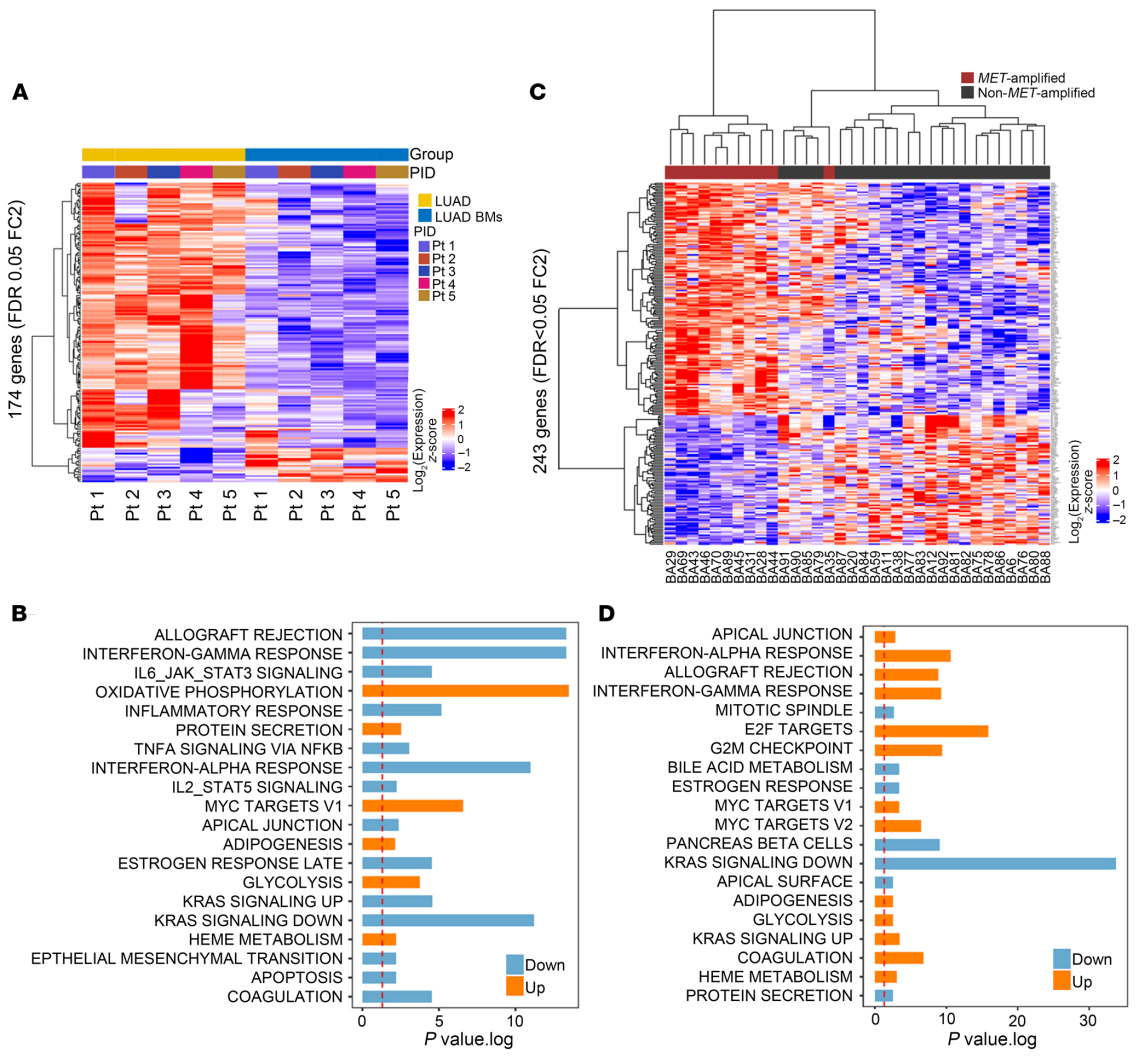
LUAD BMs have a distinct transcriptional profile compared with matched primary LUAD tumors, and *MET*-amplified BMs are distinct from non–*MET*-amplified BMs. (**A**) Heat map of 174 differentially expressed genes in 5 matched primary LUAD (yellow) and BMs (blue) (FDR < 0.05, fold change [FC] ≥ 2.0 or ≤ –2.0). (**B**) GSEA of the Hallmark gene sets from the Molecular Signatures Database (MSigDB) showing increased (orange) and decreased (blue) pathways in BMs compared with primary LUAD. The top 20 pathways are shown sorted by median rank of higher to lower (representing confidence higher to lower). (**C**) Heat map of 243 differentially expressed genes in *MET-*amplified (red) and *MET* WT (black) BMs (FDR < 0.05, fold change ≥ 2.0 or ≤ –2.0). (**D**) GSEA of the Hallmark gene sets from the MSigDB showing increased (orange) and decreased (blue) pathways in *MET-*amplified compared with non–*MET*-amplified BMs. The top 20 pathways are shown sorted by median rank of higher to lower (representing confidence higher to lower). The red dashed lines in **B** and **D** represent the threshold of what was considered significant.

To investigate transcriptomic differences between primary LUAD and LUAD BMs, we performed RNA-seq on 5 matched cases. Differential gene expression analysis identified 174 genes that were significantly differentially expressed between primary LUAD and matched BM samples (FDR = 0.05, fold change ≥ 2.0 or ≤ –2.0) (Figure 6A, ordered by group; Supplemental Figure 6, ordered by patient; Supplemental Tables 8–10). We conducted GSEA (Ensembl) using MSigDB Hallmark gene sets on the RNA-seq data from the matched samples. The top 20 pathways that were significantly up- or downregulated in LUAD BMs compared with primary LUAD are shown in Figure 6B and Supplemental Table 11. As expected (52–54), several immune-related signatures were significantly downregulated in LUAD BMs, including allograft rejection, IFN-γ response, IL-6/JAK/STAT3 signaling, inflammatory response, TNF-α signaling via NF-kB, IFN-α response, and IL-2/STAT5 signaling. The suppression of these pathways suggests diminished immune activation and cytokine signaling in the brain metastatic microenvironment, which may facilitate immune evasion and metastatic progression. The downregulation of key inflammatory and immune-mediated pathways, such as TNF-α signaling and IFN responses, indicates potential reduced proinflammatory signaling, which could be critical for the survival of LUAD cells in the brain microenvironment. To further examine immune differences between primary LUAD and BMs, we conducted immune cell subset analysis on the matched cases (Supplemental Figure 6B). In all cases, the microenvironment and immune score as well as specific immune cell types, including B cells and dendritic cells, were significantly reduced in the LUAD BMs compared with primary LUAD, indicating that BMs exhibit immune-tolerant characteristics.

Previous studies, primarily in melanoma and breast BMs, demonstrated that oxidative phosphorylation (OXPHOS) is commonly used in BMs (55, 56). As expected, OXPHOS was among the most significantly upregulated pathways in LUAD BMs; however, there was also a smaller but significant increase in glycolysis. Additionally, the upregulation of Myc targets could further indicate metabolic adaptation in the brain microenvironment. These pathway enrichment results were confirmed through fast GSEA (fGSEA; classical GSEA algorithm) (Supplemental Figure 7A and Supplemental Table 12).

### MET-amplified BMs have a distinct transcriptomic profile and immune landscape from non–MET-amplified BMs.

We next sequenced *MET*-amplified (*N* = 11) versus non–*MET*-amplified (*N* = 23) LUAD BMs and identified 243 genes that were significantly differentially expressed between these groups (Figure 6C and Supplemental Tables 13–15). Notably, a single *MET*-amplified case with the lowest amplification (MET/CEP = 2.15) clustered with the non–*MET*-amplified cases. Ensembl GSEA and fGSEA of the BM cases showed significant upregulation or modulation of immune-related processes (IFN-α and IFN-γ responses, allograft rejection, IL-6/STAT3 signaling, IL-2/STAT5 signaling, and TNF-α signaling via NF-kB), cell cycle regulation and proliferation (E2F targets, G_2_M checkpoint, mitotic spindle, Myc targets, KRAS signaling, and mTORC1 signaling), metabolic pathways (adipogenesis, glycolysis, and heme metabolism), pathways involved in EMT (apical junction, apical surface, and EMT), and coagulation pathways in *MET-*amplified BM compared with non–*MET*-amplified BM cases (Figure 6D, Supplemental Figure 7B, and Supplemental Tables 16 and 17).

We have previously shown that the EMT transcription factor TWIST1 is a downstream target of the HGF/MET pathway, is required for MET tumorigenesis, and mediates MET TKI resistance (47, 57, 58). In support of its relevance in BMs, in the BM tumor microenvironment (TME), astrocytes have been shown to induce *TWIST1* in BMs, leading to chemoresistance (59), and a prior study reported TWIST1 mRNA and protein expression in approximately 70% of BMs across breast, lung, kidney, and colon cancers as well as increased TWIST1 mRNA in a paired primary lung/BM (60). Given that we observed modulation of pathways involved in EMT, we evaluated whether TWIST1 expression would be higher in our *MET*-amplified BM cases compared with non–*MET*-amplified cases. We performed TWIST1 IHC in a subset of BM cases with available tissue. TWIST1 was detected in 55% of the *MET*-amplified cases compared with only 21% of *MET* WT BM cases (*P* = 0.047) (Supplemental Figure 8). These findings extend prior reports of TWIST1 involvement in BMs and support its association with MET pathway activation.

To further validate these findings, we utilized the Caris dataset to assess distinct immune cell populations and immune-oncology (IO) marker expression in *MET*-amplified, *MET*-altered (mutant and/or amplified), and non–*MET*-amplified/altered BMs. We first examined the expression of several IO markers and found that programmed cell death protein 1 (PD-1) and PD-L1 were significantly increased in both *MET*-altered and -amplified BMs (Supplemental Figure 9A and Supplemental Figure 10A). The increased PD-L1 expression was confirmed by PD-L1 IHC (22C3 pharmDx; 50% *MET* WT vs. 80% *MET*-altered, *P* < 0.001; data not shown). PD-L2 was also elevated in both groups but reached statistical significance only in the *MET-*altered BM group (Supplemental Figure 9A). We did not observe a significant increase in either the IFN-γ or T cell inflamed signature in this dataset when we compared either the *MET*-altered or -amplified cohorts to the non–*MET*-amplified/altered BM cohort (Supplemental Figure 9B and Supplemental Figure 10B). Conversely, in *MET*-altered BMs compared with non–*MET*-altered BMs, M1 macrophages were significantly elevated, whereas NK cells were significantly reduced (Supplemental Figure 9C). No differences were observed in other immune subsets, such as B cells, M2 macrophages, monocytes, neutrophils, CD4^+^ and CD8^+^ T cells, Tregs, or dendritic cells, between the groups. Similar trends were observed in *MET-*amplified BMs, with a significant increase in M1 macrophages and a reduction in M2 macrophages. Additionally, NK cells and CD4 T cells were also decreased in *MET*-amplified BMs (Supplemental Figure 10C). Together, these findings suggest a shift in the immune landscape toward a less immunosuppressive microenvironment in *MET*-driven BMs, characterized by altered immune cell composition and elevated immune checkpoint markers, potentially contributing to an inflamed phenotype in these tumors.

### Lung cancer patients with MET-amplified BMs have poor OS.

**Figure 7:**
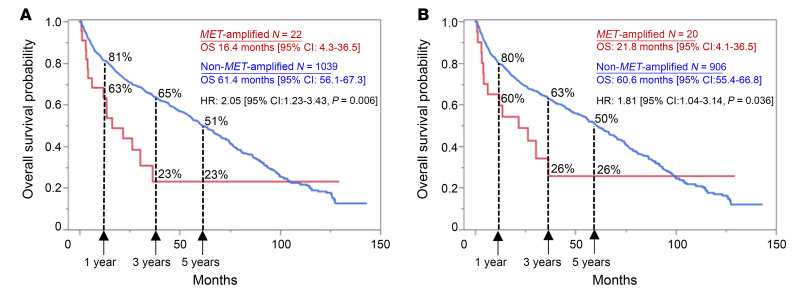
Patients with NSCLC *MET-*amplified BMs have worse OS. Kaplan-Meier survival analysis showing OS in months from initial diagnosis in patients with NSCLC BMs stratified by *MET* amplification (red line) versus no amplification (blue line). Median OS in months, HR, and CI were calculated. (**A**) All patients; (**B**) all patients excluding those with *EGFR* mutations. 1-, 3-, and 5-year survival rates are indicated.

Finally, we asked if the presence of a *MET* amplification in LUAD BMs had any prognostic significance. We analyzed OS from the time of initial lung cancer diagnosis in patients with *MET*-amplified BMs compared with those with non–*MET*-amplified BMs using data from the Caris dataset. Our findings demonstrate that patients with *MET-*amplified BMs (*N* = 22) exhibit significantly poorer OS compared with those without *MET* amplification (*N* = 1,039) (Figure 7A). At 1 year, the survival rate for patients with *MET*-amplified BMs was 63%, decreasing to 23% at both 3 and 5 years. In contrast, patients without *MET* amplification had higher survival rates, with 81% at 1 year, 65% at 3 years, and 51% at 5 years. Median OS was 16.4 months in the *MET*-amplified cohort and 61.4 months in the non–*MET*-amplified cohort (HR: 2.05; *P* = 0.006). This 3.7-fold difference in OS highlights the aggressive nature of *MET*-amplified tumors, which may drive a more rapid progression and poorer prognosis, particularly after BMs occur. Of note, this difference was still significant when patients with an *EGFR* mutant and co-occurring *MET* amplification were excluded (Figure 7B). The significantly shorter OS in patients with *MET*-amplified BMs underscores the aggressive nature of MET-driven BMs and suggests a need for novel therapeutic strategies targeting MET to improve outcomes for this patient subgroup.

## Discussion

Advances in targeted therapies and immunotherapy have dramatically improved the management of NSCLC, leading to better control of extracranial disease and prolonged survival. These agents have transformed the treatment landscape, allowing patients with NSCLC to live longer with controlled systemic disease. However, as survival increases, more patients develop BMs over the course of their disease. Treatment options for BMs include stereotactic radiosurgery, whole-brain radiation therapy, surgery in select cases, and systemic therapies with CNS penetration, such as osimertinib for *EGFR* mutant NSCLC and alectinib or lorlatinib for *ALK*-rearranged disease. Despite these advances, BMs remain a major clinical challenge for patients with lung cancer, underscoring the need for more effective CNS active therapies and prevention strategies.

In this study, we found *MET* amplification in 16% of resected LUAD BMs, even when it was not present in biopsies from extracranial sites. These amplification events were not acquired after treatment with targeted therapy and were primarily observed in BMs without targetable oncogenic drivers, representing what we believe to be a unique and potentially actionable population of patients with BMs, including those whose extracranial disease lacks a defined oncogenic driver. Importantly, several studies have demonstrated the CNS activity of the FDA-approved MET TKIs capmatinib and tepotinib as well as their respective efficacy against *MET*-amplified NSCLC (31, 35–38). Thus, identification of *MET* amplifications in BMs of NSCLC could expand the treatment options available to these patients, even when the primary tumor is MET negative. This study also reveals several limitations of the current approach used to detect molecular alterations. Prior studies examining BM-specific or -enriched alterations were dependent upon NGS technologies, which may greatly underestimate amplification rates given the need for higher cutoffs and strict algorithms to account for aneuploidy in copy number determination. In our study, we found a statistically significant increase in *MET* amplification in BMs compared with primary NSCLC or non-BM sites using the Caris NGS platform; however, the absolute percentage was significantly lower than what was observed utilizing FISH. Similarly, we previously found a statistically significant increase in *MET* amplification in BMs compared with primary lung tumors using Foundation Medicine’s dataset (4.4% [133/3,035] vs. 2.3% [170/7,277]) (*P* < 0.0001) (45). Interestingly, prior studies have demonstrated that a much lower level of *MET* amplification is needed to predict response to MET-targeted therapy when measured by FISH (MET/CEP7 ≥ 4) rather than by NGS (GCN ≥ 10) (32–34), and a range of amplification ratios have been reported to predict response to MET TKIs when detected by blood-based ctDNA assays (36, 61, 62). Despite the approval of multiple MET TKIs and other MET-directed targeted therapies in late-phase trials, the gold standard for detecting *MET* amplification in the clinic is still widely debated (18). Our findings suggest that the standard NGS approach is inadequate.

This study, along with several previously published studies, reinforces the notion that molecular testing performed on extracranial tissue is often a poor predictor of potential targetable alterations in the CNS. Prior studies demonstrated that BM-specific *HER2* amplification is found in patients with breast cancer who have HER2-negative extracranial disease (43, 63). Similarly, BM-specific copy number alterations have been identified in patients with lung cancer (44). As more BM-specific targetable alterations are identified, there is a critical need for better detection of BM-specific or -enriched alterations. Prior studies utilizing blood-based ctDNA-based assays have shown only modest performance in detecting BM-enriched or -specific alterations; some studies have suggested a better diagnostic yield from the use of relatively invasive lumbar punctures to obtain cerebrospinal fluid–derived (CSF-derived) ctDNA for detecting CNS-specific alterations (49–51). Previous studies focused primarily on patients with LUAD who had leptomeningeal disease showed that *MET* amplification is detectable and often present in the CSF, even in *EGFR* WT patients (64–66). Of note, in our current study, BMs were associated with an increased likelihood of having a *MET* amplification or *MET* mutation by ctDNA. This suggests that blood-based ctDNA assays may be capable of detecting a significant fraction of *MET* amplification in BMs; however, this needs to be confirmed in a prospective study. A limitation of our study is the limited overlap between the ctDNA and FISH cohorts, with only 4 patients having both blood and brain tissue available, which precluded a direct comparison of *MET* status between blood and tissue. Alternatively, radiomic approaches have been used on CT images of pulmonary nodules to predict mutational subtypes in NSCLC (67) and on brain MRI images to detect mutational subtypes in glioblastoma (68, 69) and *EGFR* or *KRAS* mutations in lung cancer BMs (70–74). It is possible that a radiomic approach could be used to detect BM-specific alterations such as *MET* amplifications.

In contrast to published data showing the that the HGF/MET pathway promotes an extracranial immunosuppressive TME (75–83), our findings suggest that *MET*-amplified BMs have a more inflammatory transcriptional signature, along with increased expression of PD-1 and PD-L1, and significant upregulation or modulation of immune-related processes (IFN-α and IFN-γ responses, allograft rejection, IL-6/STAT3 signaling, IL-2/STAT5 signaling, and TNF-α signaling via NF-kB) compared with non–*MET-*amplified BM cases. Of note, the IL-6/JAK/STAT3 pathway was increased in *MET*-amplified BM compared with non–*MET*-amplified BM cases. Prior studies in extracranial disease have suggested that MET/STAT3 signaling is associated with immune evasion via M2 macrophage polarization, myeloid-derived suppressor cell (MDSC) expansion, and increased cancer-associated fibroblast signaling leading to MDSC migration (75, 84–88). Although we did not observe any evidence of an increased activated T cell population, there was a notable increase in M1 macrophages and a corresponding decrease in M2 macrophages, suggesting a potentially less immunosuppressive TME. As such, it is possible that utilizing MET inhibitors for *MET*-amplified BMs may have the unintended effect of suppressing immune responses. It is also possible that this inflammatory brain microenvironment could make *MET*-amplified BMs more sensitive to immunotherapy; however, these hypotheses require both preclinical and clinical validation. A future direction is also to investigate whether upregulation of STAT3 signaling in *MET*-amplified BMs leads to a more immunosuppressive TME. Notably, previous studies of molecularly unselected LUAD BM patients demonstrated modest but consistent CNS activity of the anti–PD-1 agent pembrolizumab or the combination of the anti–CTLA-4 ipilimumab and the anti–PD-1 agent nivolumab (89–91).

Interestingly, we found unexpected metabolic differences in *MET*-amplified BMs. While previous studies have demonstrated that melanoma and breast cancer BMs primarily utilize OXPHOS regardless of the metabolic pathways used extracranially (55, 56), we observed increased glycolysis in our *MET-*amplified BMs. We saw increased OXPHOS in our LUAD BM versus LUAD lung samples consistent with these prior studies. However, it appears that *MET*-amplified BMs, which utilized primarily glycolysis, are distinct from non–*MET-*amplified BMs. Interestingly, prior cell line studies have demonstrated that MET is a major driver of glycolysis, at least extracranially (92–94). Notably, several glycolysis inhibitors, such as 2-deoxy-glucose (2DG) and PFK158, with CNS penetration, have been tested in early-phase trials (95, 96). Additionally, newer 2DG analogs, such as WP1122, have been developed with an increased half-life and enhanced blood-brain barrier penetration (97). Finally, newer agents, such as BPM31510 (ubidecarenone), which induced a metabolic switch from glycolysis to OXPHOS, have shown promising results preclinically and in early-phase trials as well (98–106). It is possible that these glycolytic inhibitors could be another therapeutic strategy for targeting *MET*-amplified BMs. Furthermore, as increased lactate in the TME due to MET-driven glycolysis extracranial appears to contribute to an immunosuppressive TME (75), it is possible that combinations examining glycolytic inhibitors with immunotherapy agents may be effective against *MET*-amplified BMs as well.

Mechanistically, we have previously shown that the EMT transcription factor TWIST1, which has been implicated in BMs (59, 60), is essential for MET-driven tumorigenesis (47, 57, 58), is regulated by the HGF/MET signaling axis (47), and can confer resistance to MET TKIs (47). TWIST1 has also been shown to suppress apoptosis by downregulating proapoptotic factors (e.g., BIM) (107). These findings suggest that TWIST1 may mediate a dual prosurvival and prometastatic program through both suppression of apoptosis and induction of EMT in an HGF/MET-dependent manner. Our findings that TWIST1-positive BMs were more likely to be *MET* amplified also suggests a mechanistic link between TWIST1 expression and activation of the MET pathway in metastatic progression to the brain. A future direction is to further evaluate the functional role of TWIST1 in *MET*-amplified BMs.

Our studies suggest that the hypoxia-inducible factor 1-α (HIF-1α) pathway may be important for BMs as VHL mutations were only found in BMs and not primary LUAD. Interestingly, we also found that VHL mutations were exclusively present in *MET* mutant BMs but absent in *MET*-amplified BMs. Prior studies have shown that hypoxia increases MET expression via HIF-1α and that MET increases HIF-1α protein levels (108–110). Furthermore, coexpression of high MET and HIF-1α has been reported in breast cancer (111). It is possible that some MET mutational variants are unable to sufficiently stabilize HIF-1α; thus, loss of VHL is necessary to drive HIF-1α protein expression. A future direction is to examine whether the HIF-1α transcriptional program is activated in *MET* mutant versus *MET*-amplified versus *MET* WT BMs.

While this study provides important insights into *MET* amplification in LUAD BMs, several limitations should be acknowledged. First, although our findings are based on well-annotated human specimens and validated in a large, independent patient cohort, the observational nature of clinical tissue-based research limits our ability to experimentally test mechanistic hypotheses. Second, not all patient cohorts utilized the same assay for detection of *MET* amplification (FISH vs. NGS) nor contained the same granularity of patient data, which made it difficult to integrate datasets. Third, our current study focuses on detectable genetic alterations in MET leading to its overexpression and activation, but a recent study has suggested that upregulation of mesothelin (MSLN) is a nongenomic mechanism of MET activation in BMs (112). Interestingly, our RNA-seq data showed increased *MSLN* mRNA in BM versus primary LUAD samples, but it was decreased in *MET*-amplified BMs compared with non–*MET*-amplified BMs, suggesting that *MET* amplification and high *MSLN* mRNA expression are mutually exclusive. Finally, while we identified immune, metabolic, and mutational changes associated with *MET* alterations, the functional consequences of these changes have not yet been directly tested in preclinical model systems. Future studies utilizing genetically engineered mouse models, in vivo metastases models, organotypic brain slice cultures, and targeted functional perturbations are essential to define the mechanistic role of *MET* amplification and its downstream signaling networks in LUAD BM biology. Nonetheless, these findings provide a critical foundation for understanding *MET*-altered BMs and offer strong rationale for development of targeted MET therapies in patients with LUAD BMs.

In conclusion, the increasing incidence of BMs underscores the need for deeper characterization to uncover novel therapeutic strategies. Our findings identified a significant enrichment of *MET* amplification in oncogene driver–negative LUAD BMs, independent of prior targeted therapy, indicating that this was not merely a consequence of acquired resistance. Additionally, our study found a distinct molecular and transcriptomic landscape in LUAD BMs, shaped by unique immune and metabolic adaptations and induction of an EMT program that distinguished primary LUAD from LUAD BMs as well as *MET*-altered BMs from non–*MET*-altered BMs. Furthermore, patients with *MET*-amplified BMs had significantly worse survival. Finally, our findings suggest that targeting *MET* amplification could present a therapeutic opportunity for a large subset of patients with LUAD BMs. Prospective trials validating ctDNA for MET detection and combining MET TKIs with glycolysis inhibitors or immunotherapy are warranted.

## Methods

### Sex as a biological variable.

Our study cohort included both male and female patients with LUAD; the sex distribution is reported in Tables 1–3. In the in vivo experiment, only female mice were used. This choice was based on prior findings from our group demonstrating more consistent tumor establishment in female mice. While this approach reduced biological variability, we acknowledge the limitation of using a single sex and will incorporate both male and female animals in future studies.

### Statistics.

For the in vivo PDX experiment, a 2-sided Student’s *t* test was performed on the final tumor volume between the control and capmatinib treatment groups. Bioluminescent imaging data from the in vivo experiment were analyzed using the Mann-Whitney test. A 2-sided Fisher’s exact test was used to determine significant differences in *MET* amplification by tumor type, in mutations found in primary LUAD versus BMs and *MET*-altered LUAD BMs versus non–*MET*-altered BMs. *P* values were adjusted for an FDR of 0.05 using the Benjamini-Hochberg method. *P* values of less than 0.05 were considered significant. A Fisher’s exact test was also performed when evaluating specific gene variants shown in Supplemental Figures 2, 3, and 5 and to compare the frequency of ctDNA *MET* alterations, including *MET* amplifications and mutations, in individuals with and without BMs. A χ^2^ test was used to determine if there was a statistically significant difference in the rate of metastatic disease to the brain in the focally *MET*-amplified versus nonfocally *MET*-amplified BMs and for differences in patient characteristics in Tables 1–3. Survival curves were estimated using the Kaplan-Meier method.

### Study approval.

This study was conducted under University of Pittsburgh IRB protocol 12070229 and STUDY19110031. Patient tissue to generate the PDX model was obtained from patients undergoing standard-of-care craniotomy after informed consent under University of Pittsburgh IRB protocol 19080321. Animal studies were approved and conducted under University of Pittsburgh IACUC protocol 21089597.

### Data availability.

RNA-seq data FASTQ files (*N* = 40) were deposited in the National Center for Biotechnology Information Sequence Read Archive database (accession number PRJNA1129590). Additional details regarding data and protocols that support the findings of this study are available from the corresponding authors upon request. Values for all data points in the graphs are provided in the Supporting Data Values file. All remaining materials and methods are described in Supplemental Methods.

## Author contributions

Conception and design, methodology, and funding acquisition: TFB, SD, and LPS. Data acquisition: TFB, SD, MAV, EM, AS, SB, AGD, PD, JJ, VK, JX, and LPS. Data analysis and interpretation of data: TFB, SD, AC, JX, URC, RB, CT, and LPS. Writing (original draft): LPS and TFB. Writing (review and editing): TFB, SD, AC, EM, MAV, AS, SB, AGD, PD, JJ, VK, AP, JX, MO, MJG, SA, URC, RB, CT, and LPS.

## Funding support

This work is the result of NIH funding, in whole or in part, and is subject to the NIH Public Access Policy. Through acceptance of this federal funding, the NIH has been given a right to make the work publicly available in PubMed Central.

- National Cancer Institute (R01 CA244270-01A1 to TFB and LPS).
- Department of Defense LCRP-IITRA (W81XWH-22-1-0350 to TFB and LPS).
- National Cancer Institute (P50CA090440 to TFB, LPS, and SD).
- American Cancer Society Research Scholar Award (RSG TBG – 132939 to TFB, LPS, and SD).
- The University of Pittsburgh Institute for Precision Medicine (to TFB and LPS).
- Libby’s Lungs (to LPS).
- We Wish Foundation (to TFB).
- National Cancer Institute grant (P30CA047904) awarded to the UPMC Hillman Cancer Center–supported research reported in this publication using the Biostatistics Facility (RRID: SCR025355), Cancer Bioinformatics Services (RRID: SCR025356), Cancer Genome Facility (RRID: SCR025357), In Vivo Imaging Facility (RRID: SCR025360), and Animal Facility (RRID: SCR025152).
- Lung Cancer Research Foundation and MET Crusaders Research Grant on MET-Driven Lung Cancer (to TFB and LPS).

## Supplementary Material

Supplemental data

Supplemental tables 8-17

Supporting data values

## Figures and Tables

**Table 1:**
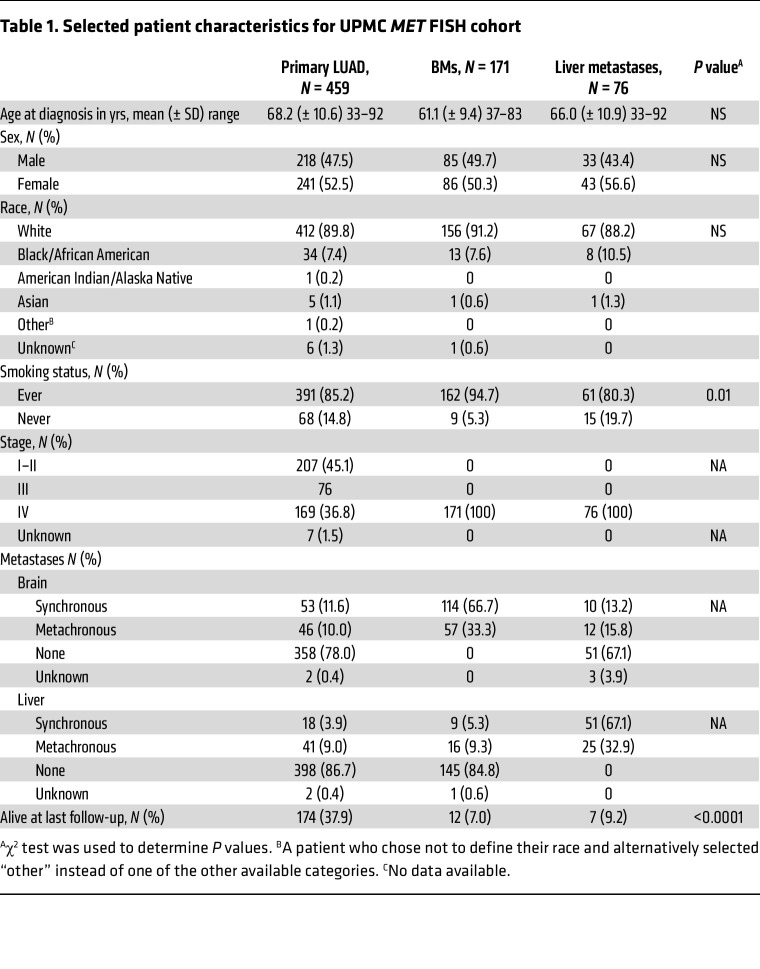
Selected patient characteristics for UPMC *MET* FISH cohort

**Table 2:**
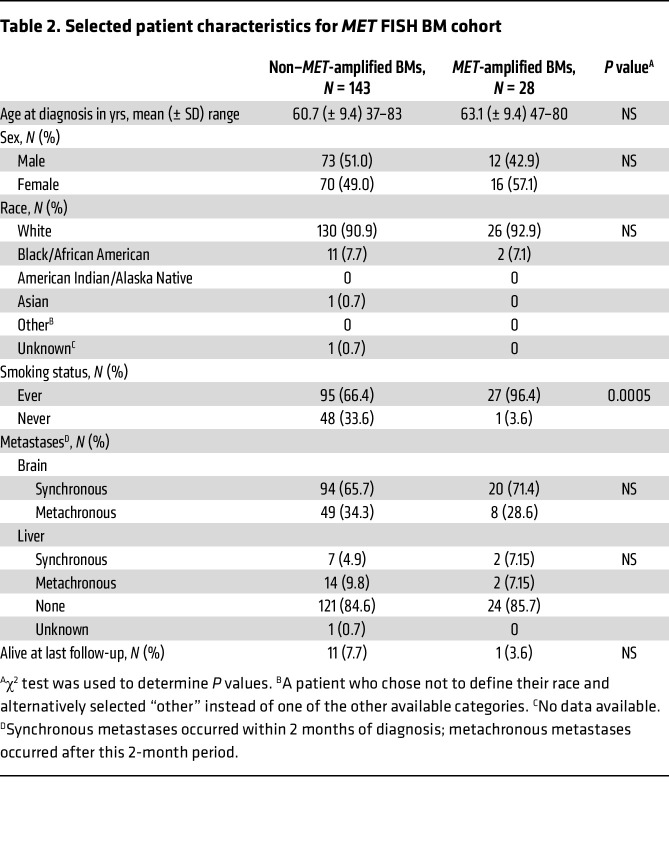
Selected patient characteristics for *MET* FISH BM cohort

**Table 3:**
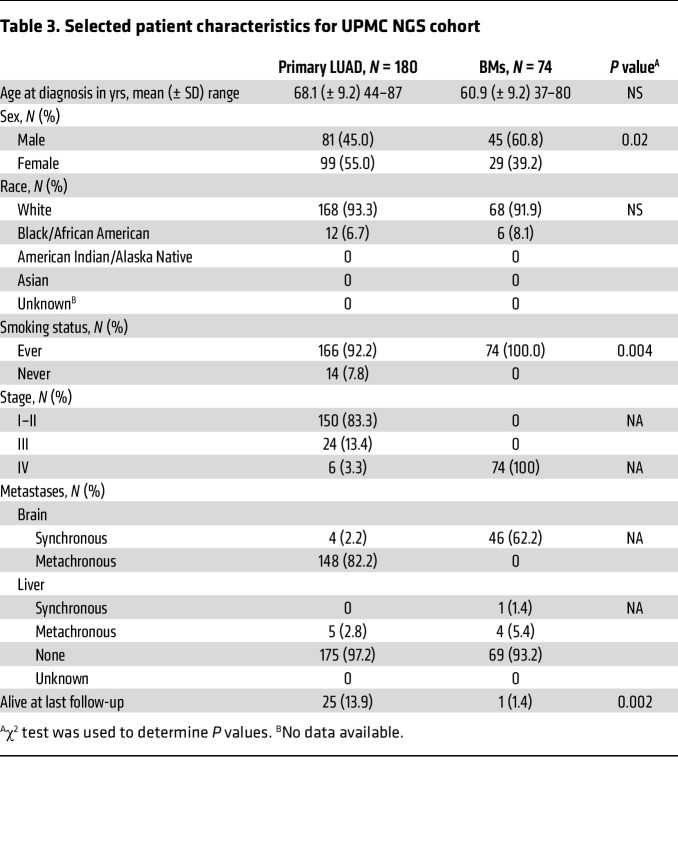
Selected patient characteristics for UPMC NGS cohort

## Version 1 12/18/2025

In-Press Preview

## Version 2 02/16/2026

Electronic publication

## Version 3 02/16/2026

edits made to figure labels
