# Supplementary material for: MET alterations are enriched in lung adenocarcinoma brain metastases, defining a distinct biologic subtype
Source: J Clin Invest. 2025 Dec 18;136(4):e194708. doi: 10.1172/JCI194708 (PMC12904726; doi:10.1172/JCI194708)
Supplement: Supplemental data [file jci-136-194708-s011.pdf]

Supplemental Data File:

Supplemental Methods

Supplemental Figures 1-10 and Legends

Supplemental Tables 1-7

Supplemental References

Supplemental Methods

Generation of MET-amplified BM PDX 16-16. BM PDX 16-16 was established from a patient undergoing standard-of-care craniotomy after informed consent to a University of Pittsburgh Institutional Review Board (IRB)-approved protocol allowing for generation of PDX and use of medical history as previously described (1).

MET amplification analysis by FISH. FISH analysis of *MET* amplification was performed on FFPE tumor sections using the dual-color MET SpectrumOrange/centromere 7 (CEP7) SpectrumGreen probe (Vysis, Downers Grove, IL, USA) and paraffin pre-treatment reagent kit (Vysis, Inc.) (2). Quality control was ensured through the inclusion of positive and negative control samples in a Clinical Laboratory Improvement Amendments (CLIA)-certified laboratory. At least 60 cells were scored for each case. *MET* gene amplification was defined as a MET/CEP7 ratio ≥ 2. Focal *MET* amplification was defined as clusters of 5-10 cells (< 25%) of the tumor area with *MET* amplification.

Patient-derived xenograft (PDX) experiment. Female SCID mice (6-8 weeks of age; Jackson Labs, Bar Harbor, ME, USA) were subcutaneously implanted in the hind flank with a 0.5 × 0.5-mm piece of PDX 16-16 tumor in 50% matrigel. When the tumor volume reached ~200 mm^3, mice were randomly divided into the control or the capmatinib treatment group (*N* = 5-6/group). Methyl cellulose (0.25% w/v) was used as vehicle control. Capmatinib (5 mg/kg; MedChem Express, NJ, USA) was dissolved in 0.25% w/v methyl cellulose and administered by oral gavage 5 times a week for 4 weeks. Tumor size was measured twice a week with digital calipers, with the volume calculated as [1/2(length × $width^2$)]. All procedures were approved by the University of Pittsburgh Institutional Animal Care and Use Committee (IACUC), adhering to all guidelines and regulations. No systemic toxicities or changes in body weight were observed with experimental treatment.

Intracardiac injection mouse model. H1993 cells were purchased from American Type Culture Collection (ATCC) and grown in recommended media and luciferase labeled as described previously (3). Luciferase-labeled H1993 LUAD cells were injected intracardiacally into SCID mice (Jackson Labs) to track metastatic dissemination. Cells were cultured under standard conditions and harvested at ~80% confluency and resuspended in sterile PBS at a concentration of $1X10^5$ cells/100µl. Mice were anesthetized using isoflurane and placed in the supine position. A small midline incision was made to expose the ribcage and facilitate needle placement. Using a 29-gauge needle, the cell suspension was injected into the left ventricle of the heart. Successful injection was confirmed

by the appearance of bright red arterial blood in the syringe hub and immediate systemic perfusion of the luciferase-labeled cells, as visualized by *in vivo* bioluminescent imaging (IVIS Lumina XR System; PerkinElmer, Shelton, CT, USA) after D-luciferin (30mg/ml; i.p.) administration. Mice were imaged weekly to monitor metastatic progression. Bioluminescent signal intensity was quantified using the Living Image Software (PerkinElmer), with regions of interest (ROIs) defined over the head. Treatment with vehicle or capmatinib (using the same dosing schedule as above) was initiated upon detection of a predefined signal threshold in the head, indicating early metastatic colonization. Only mice demonstrating a confirmed *ex vivo* brain signal at endpoint were included in the study. All procedures were conducted in accordance with the University of Pittsburgh IACUC guidelines.

Immunohistochemistry. IHC was performed for MET using the rabbit monoclonal anti-c-MET antibody (#08866856001/clone SP44; Ventana/Roche, Tucson, AZ, USA) and for TWIST1 using the mouse monoclonal anti-Twist antibody (ab50887/clone Twist2C1a; Abcam, Cambridge, MA, USA) in human FFPE 4μm thick tissue sections. MET and TWIST1 protein expression was quantified by a board-certified pathologist (S.D). Staining intensity was scored as 0 (no staining), 1+ (weak), 2+ (moderate), or 3+ (strong). An H-score was calculated for each sample by multiplying the staining intensity by the percentage of positive tumor cells, resulting in a score range from 0 to 300. A TWIST1 H-score of >10 was considered positive. For the xenograft FFPE tissue, total MET and pMET IHC was performed using clones D1C2 (#8198; Cell Signaling Technology, Beverly, MA ,USA) and D26 (#3077, Cell Signaling Technology), respectively.

UPMC patient cohort for MET FISH analysis. Consecutive patients with LUAD who underwent surgical procedures at the University of Pittsburgh Medical Center (UPMC) (459 primary LUAD and 171 with BMs) were selected for the study; all data was obtained with approved University of Pittsburgh IRB protocols. In addition, 76 LUAD patients with liver metastases and with tissue sufficient for molecular testing were selected. No study patients received whole-brain radiation. Paired primary lung carcinoma specimens and BMs were identified in 31 patients. Clinical information including age, sex, race, smoking status, stage, and metastasis and survival data were obtained from a review of patients' electronic medical records and the UPMC Network Cancer Registry. The histologic type of each lung carcinoma was confirmed by a pulmonary pathologist (S.D.) according to 2021 World Health Organization (WHO) classification criteria. Immunohistochemistry for TTF-1 (clone 8G7G3/1; Dako, Copenhagen, Denmark, 1:50) and p40 (predilute polyclonal rabbit; Biocare Medical, Concord, CA, USA) was performed to discern difficult cases. All specimens had a tumor cellularity equal to or greater than 60%.

Synchronous metastases were defined as those occurring within 2 months of initial diagnosis, while metachronous metastases were defined as those occurring after this 2-month period.

Caris patient cohort. We identified 20,283 LUAD patients, 1756 LUAD patients with BMs, and 9966 LUAD patients with non-BMs from the Caris NGS database. Top non-BM sites included liver, bone, adrenal gland, connective tissue, and head and neck. For *MET* amplification, copy number alteration (CNA) of each exon was determined by calculating the average depth of the sample along with the sequencing depth of each exon and comparing this calculated result to a pre-calibrated value, with amplification defined as a MET GCN ≥ 6.

Real-world OS (rwOS) information was obtained from insurance claims data and calculated from patients' first cancer diagnosis to last contact. HR was calculated using the Cox proportional hazards model, and *P* values were calculated using the log-rank test.

For NGS, FFPE specimens underwent pathology review to measure percent tumor content and tumor size; a minimum of 10% of tumor content in the area for microdissection was required to enable enrichment and extraction of tumor-specific RNA and 20% for DNA. The Illumina NovaSeq 6500 was used to sequence the whole transcriptome from patients to an average of 60M reads. Raw data was demultiplexed by Illumina Dragen BioIT accelerator, trimmed, counted, PCR-duplicates removed, and aligned to human reference genome hg19 by STAR aligner. For transcription counting, transcripts per million values were generated using the Salmon expression pipeline. RNA deconvolution QuanTISeq previously reported was used to infer cell types in the tumor microenvironment (4). For NGS of DNA, NextSeq or NovaSeq 6000 platforms (Illumina, Inc., San Diego, CA, USA) were used. All variants were detected with > 99% confidence based on allele frequency and amplicon coverage, with an average sequencing depth of coverage of > 500 and an analytic sensitivity of 5%. Genetic variants identified were interpreted by board-certified molecular geneticists and categorized as pathogenic, likely pathogenic, variant of unknown significance, likely benign, or benign, according to the American College of Medical Genetics and Genomics (ACMG) standards. When assessing mutation frequencies of individual genes, pathogenic and likely pathogenic were counted as mutations.

Targeted NGS. Genomic DNA was isolated from fresh frozen tissue or FFPE tissue from a subset of cases (**Table 2**) from the UPMC patient cohort used for FISH analysis using the Puregene Tissue Kit (Qiagen, Germantown, MD, USA). For each sample, 200 ng of genomic DNA was used to generate uniquely barcoded sequencing

libraries using the Ion DNA Barcoding and Ion Xpress Template kits (Life Technologies, Carlsbad, CA, USA). Targeted NGS was performed using the Ion Torrent Hotspot Cancer Panel v.2. Variant analysis was conducted using VarScan 2 and GATK, with built-in normal tissue control sequences for comparison against the human reference genome (hg19) and to identify somatic mutations. Known benign SNPs and synonymous mutations were excluded. A pathologic score cutoff of > 0.5 was applied, and any variants below this threshold were excluded. The remaining variants were categorized as novel somatic mutations, known somatic mutations, or known benign mutations based on validation in the literature. Known benign mutations were excluded from the analysis. These variants were annotated using Vcf2maf v1.6.8 (https://github.com/mskcc/vcf2maf) with VEP cache 85 with hg19 reference genome to produce mutation allele format (MAF) files. Only variants from a pre-determined list and with 1000-g frequency of < 0.05 were kept. *METΔex14* status was verified by Sanger sequencing. Oncoplots and Co-oncoplots from MAF files were generated by maftools (5), a Bioconductor R package.

ctDNA-detected MET alterations. Individuals with biopsy-proven NSCLC and peripheral blood Guardant360 CDx assay collected between August 2014 – May 2020 (inclusive) at UPMC Hillman Cancer Center were queried for presence of BMs under a University of Pittsburgh IRB-approved protocol. Brain metastases were present if reported on brain MRI scans performed at any point prior to, or up to 3 months after, ctDNA blood collection.

RNA extraction. Total RNA was extracted from 5-μm FFPE sections (1-4 slides per sample based on tumor area) for each tumor specimen using the Quick RNA FFPE Extraction Kit (Zymo Research, Irvine, CA, USA) according to the manufacturer's protocol. Tumor cellularity was evaluated on H&E-stained slides. If tumor cellularity was less than 60%, macro-dissection was performed (7/40 cases). RNA concentration was eluted in nuclease-free water, and concentration was measured using a NanoDrop spectrophotometer (Thermo Scientific, Waltham, MA, USA) and the Qubit RNA High Sensitivity Assay Kit on the Qubit FLEX fluorometer (Thermo Scientific). RNA quality was assessed using the Agilent Bioanalyzer system and the RNA Pico Kit.

Library preparation and RNAseq. Library preparation was performed using 100 ng of input RNA and the TruSeq™ RNA Exome Kit (Illumina, San Diego, CA, USA). Quality control was assessed using the High Sensitivity NGS Fragment Analyzer Kit (Advanced Analytical Technologies, Inc., Ankeny, IA, USA) and libraries quantitated using the KAPA Library Quantification Kit (Roche). Indexed, pooled libraries were sequenced on a high-output flow cell

with a NovaSeq 6000 S2 System at the UPMC Genome Center. Forty samples were sequenced, including 35 BM samples and 5 matched primary LUAD tumors.

RNAseq data analysis. After quality control, paired-end reads were aligned against the human reference transcriptome (GRCh38) with Gencode annotation (v32) to generate transcript-level counts using Kallisto (v0.46.1) (6) following the reverse-stranded protocol, and summarized at gene-level using tximport (v1.16.0) (7). Lowly expressed genes were removed prior to statistical analysis. The read count matrix was normalized using the trimmed mean of M values (TMM) method (8), and $\log_2$-transformed. Differentially expressed genes (DEGs) were identified using limma voom with precision weights (v3.45.0) (9) and filtered at FDR-adjusted $P < 0.05$ and fold change at least 2.0. Ensemble GSEA (EGSEA) and classic GSEA were conducted using R package EGSEA (v1.28.0) (10) and fGSEA (1.16.0) (11), respectively, with Hallmark gene sets from the Human Molecular Signatures Database (MSigDB) (v7.0) (12) based on DEG results (without filtering by *P*-value or fold change) comparing primary LUAD to BMs and *MET*-amplified BMs to non-*MET*-amplified BMs. The top 20 gene sets by EGSEA or fGSEA were shown as results. Statistical analysis was conducted using R (v4.0.0) and Bioconductor (v3.11).

Digital deconvolution of RNAseq data. Cell subsets were predicted from the RNAseq data using xCell (v1.0.0) (13) with transcripts per million mapped reads (TPM) as the input, which implements both single-sample GSEA (ssGSEA) and deconvolution algorithms to compute scores. The xCell scores were then compared in primary LUAD to BMs and *MET*-amplified BMs to non-*MET*-amplified BMs using 2-sided Wilcoxon rank sum test, and filtered by FDR-adjusted $P < 0.20$. A more lenient FDR cutoff was used here for exploratory purposes, considering the scores were predicted based on signals of multiple reference genes and not individual genes.

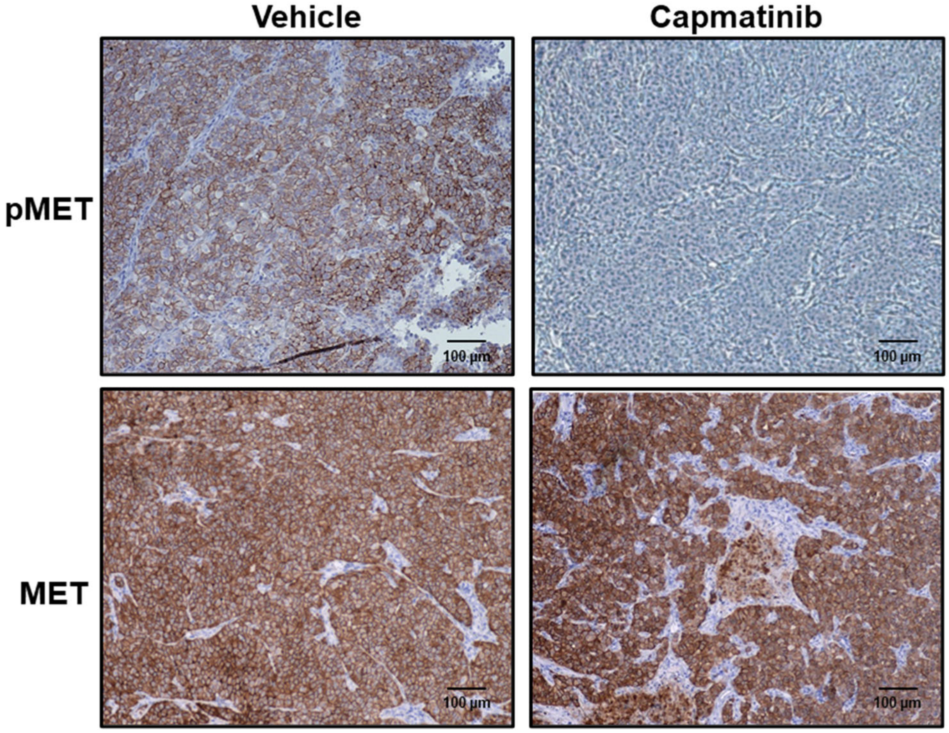

Supplemental Figure 1. Representative IHC images of pMET (top panels) and total MET (bottom panels) showing no pMET expression in 16-16 PDX tumors from mice following treatment with capmatinib. Scale bar=100 µm.

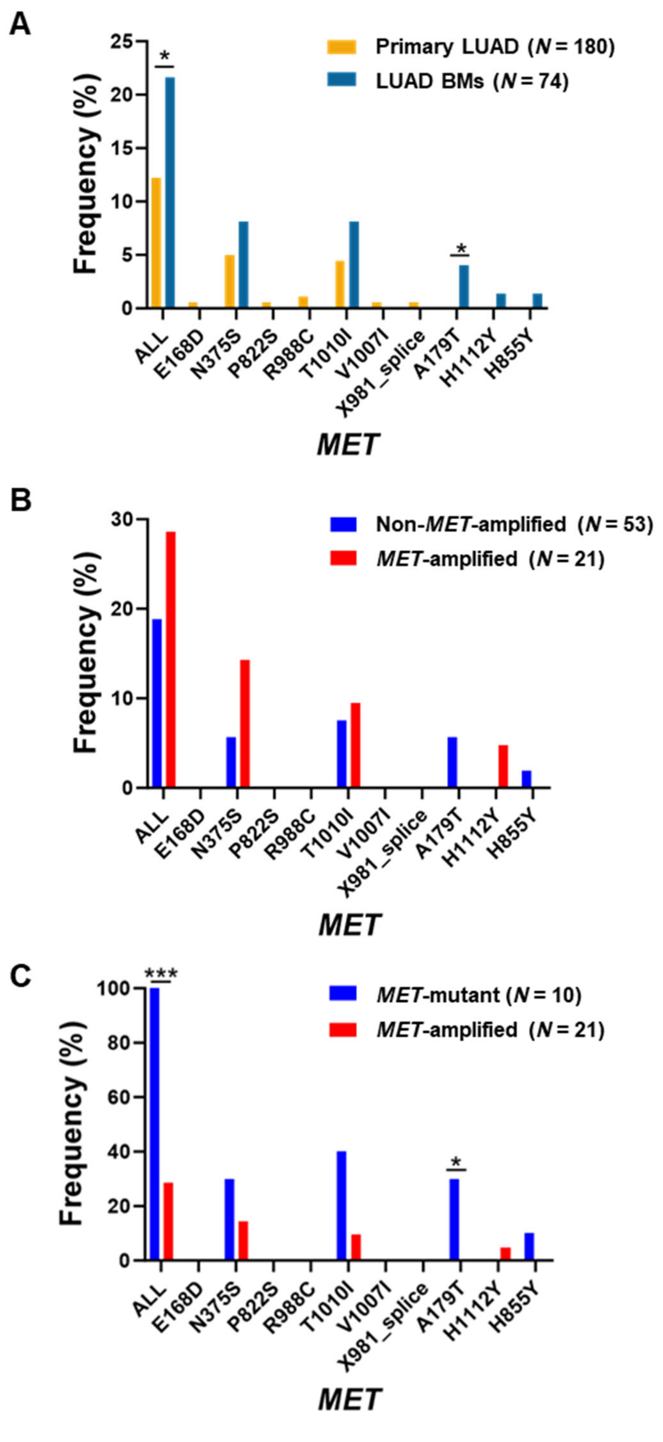

Supplemental Figure 2. Frequency of *MET* variants in primary LUAD versus LUAD BMs. **A)** Primary LUAD (yellow) versus LUAD BMs (blue); **B)** Non-*MET*-amplified (blue) versus *MET*-amplified (red); **C)** *MET*-mutant (blue) versus *MET*-amplified (red). Fisher's exact test, 2-sided; *$P < 0.05$, ***$P < 0.005$.

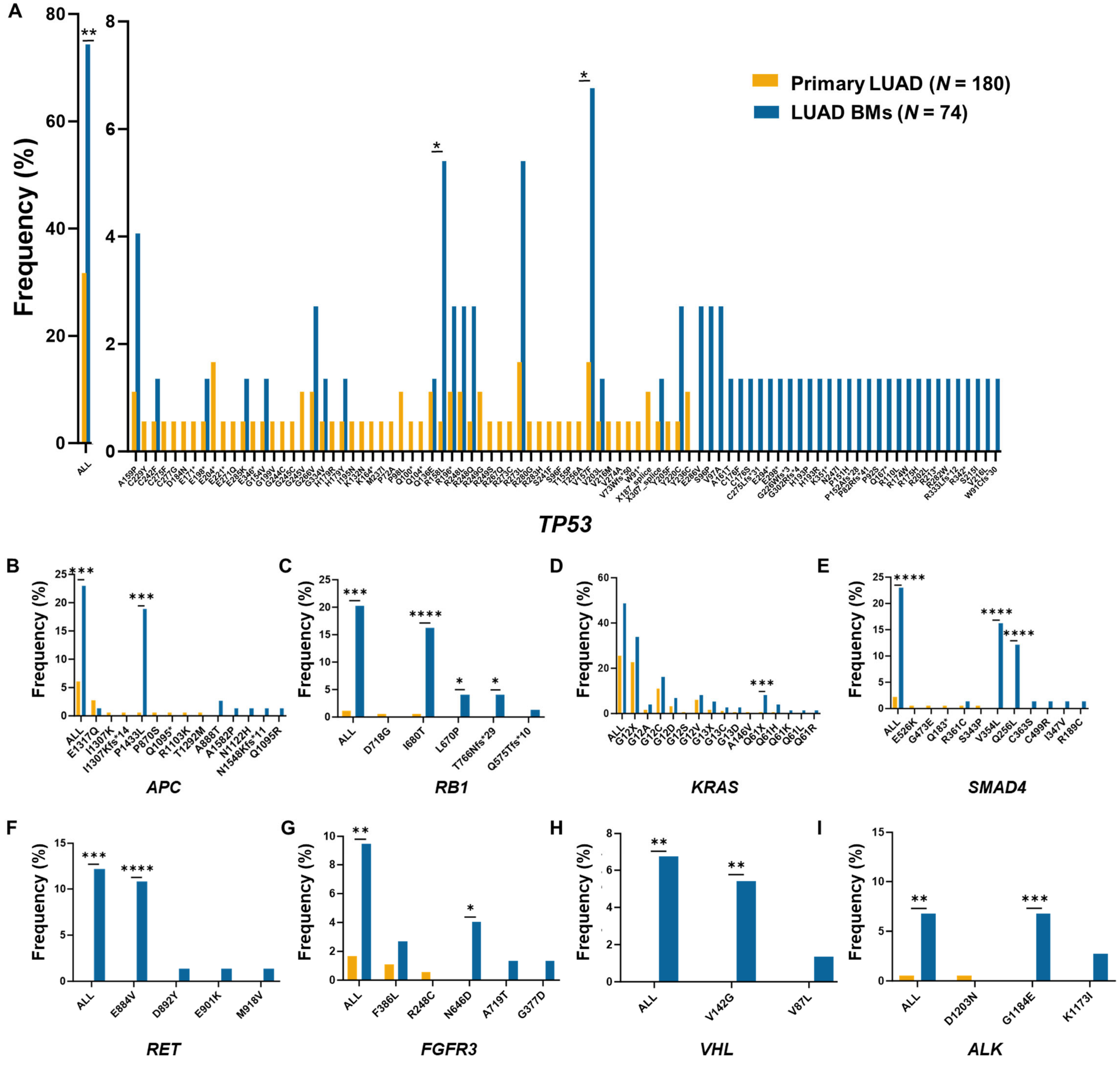

Supplemental Figure 3. Frequency of gene variants that are different in primary LUAD versus LUAD BMs.

A) *TP53*; **B)** *APC*; **C)** *RB1*; **D)** *KRAS*; **E)** *SMAD4*; **F)** *RET*; **G)** *FGFR3*; **H)** *VHL*; **I)** *ALK*. Primary LUAD (yellow); LUAD BMs (blue). Fisher's exact test, 2-sided; * $P < 0.05$; *** $P < 0.005$.

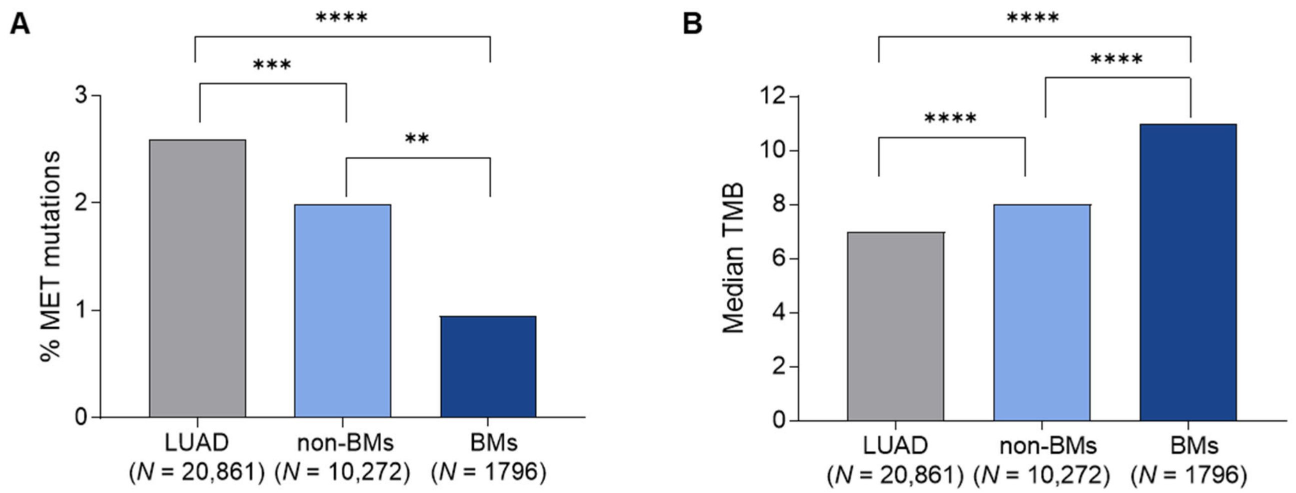

Supplemental Figure 4. MET mutations and tumor mutational burden (TMB) in primary LUAD, non-BMs, and BMs in Caris cohort. Frequency of *MET* mutations **A)** and median TMB **B)** in Caris cohort, categorized into primary LUAD, non-BM, and BM cases. The number of cases in each category is shown beneath each group. Chi-squared test, **P < 0.01; ***P<0.001; ****P < 0.0001.

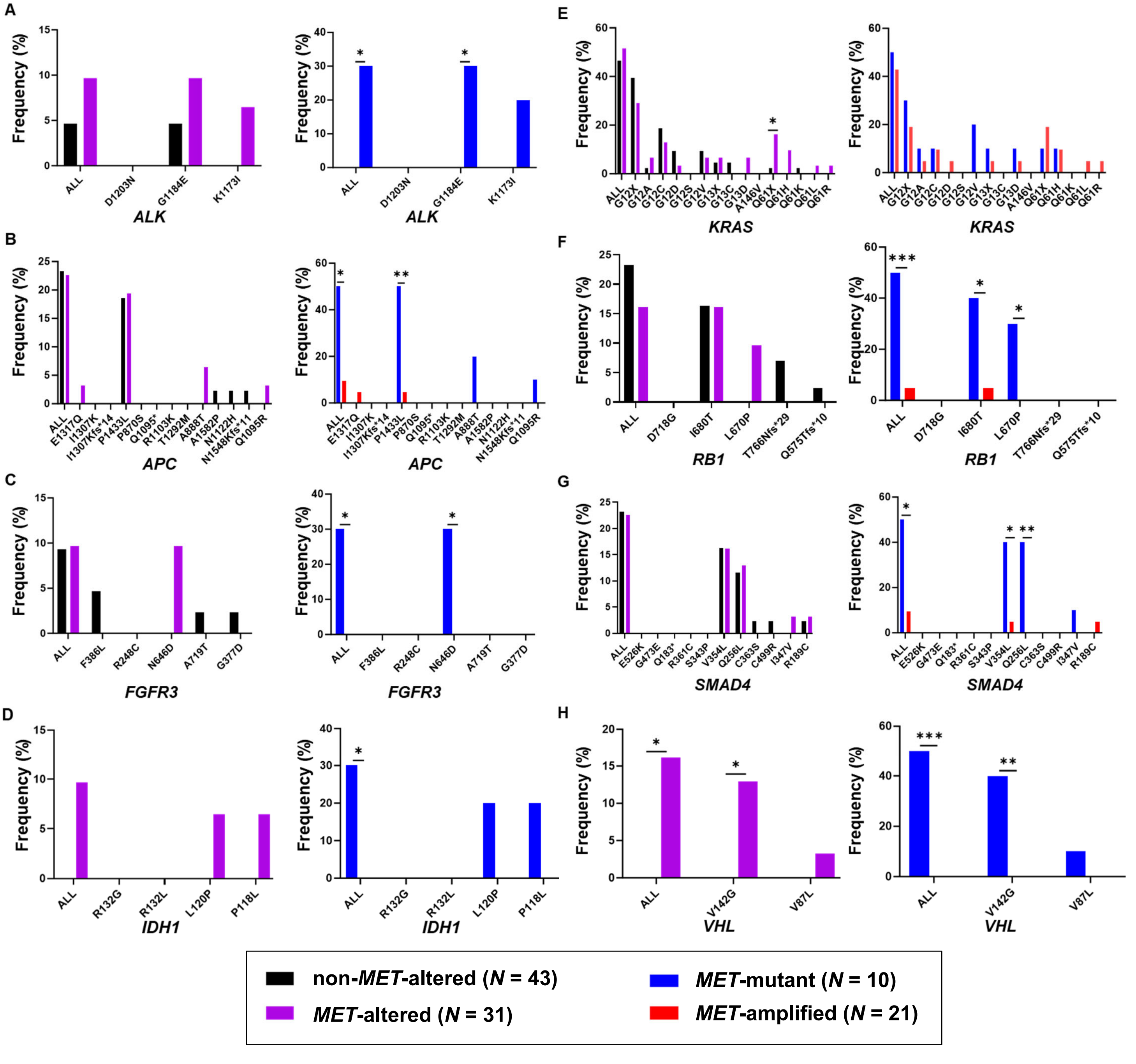

Supplemental Figure 5. Frequency of genes that are not significantly different in *MET* WT versus *MET*-altered BMs but differ by type of *MET* alteration. A) *ALK*; **B)** *APC*; **C)** *FGFR3*; **D)** *IDH1*; **E)** *KRAS*; **F)** *RB1*; **G)** *SMAD4*; **H)** *VHL1*. Fisher's exact test, 2-sided: * $P < 0.05$; ** $P < 0.01$; ***$P < 0.005$.

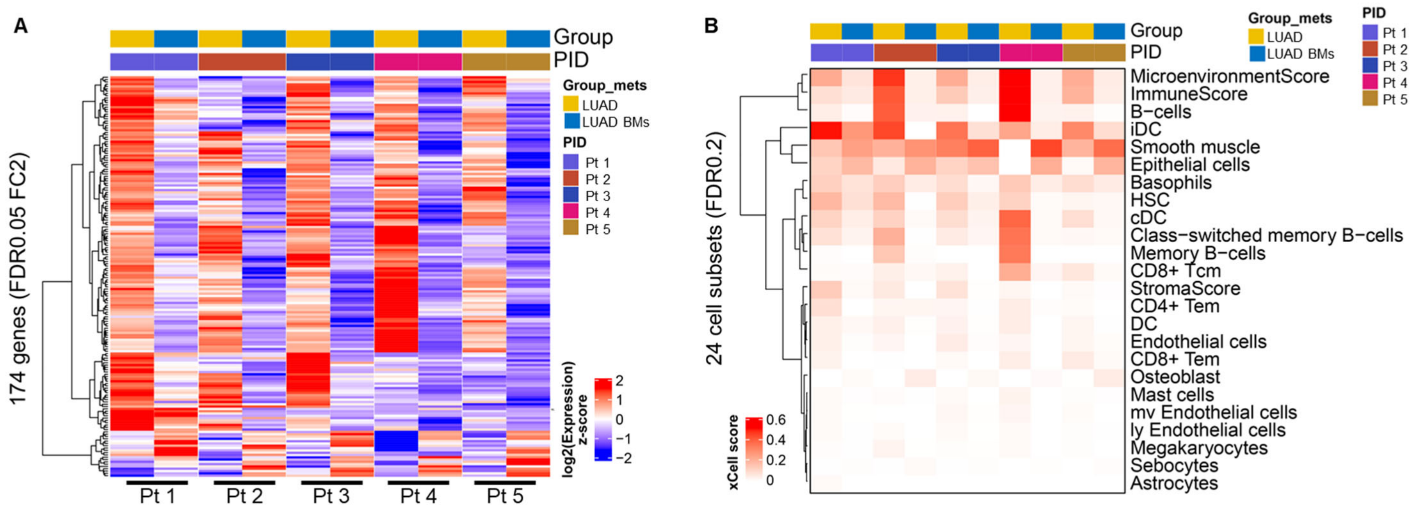

Supplemental Figure 6. RNA expression profile ordered by matched sets. A) Heatmap of 174 differentially expressed genes in 5 matched primary LUAD (yellow) and BMs (blue) ordered by matched patient set. (FDR < 0.05, fold change ≥ 2.0 or ≤ -2.0; FC2). **B)** Heatmap of 24 digitally deconvoluted cell subsets in the 5 matched primary LUAD (yellow) and BMs (blue) ordered by patient. PID, patient ID; Pt, patient.

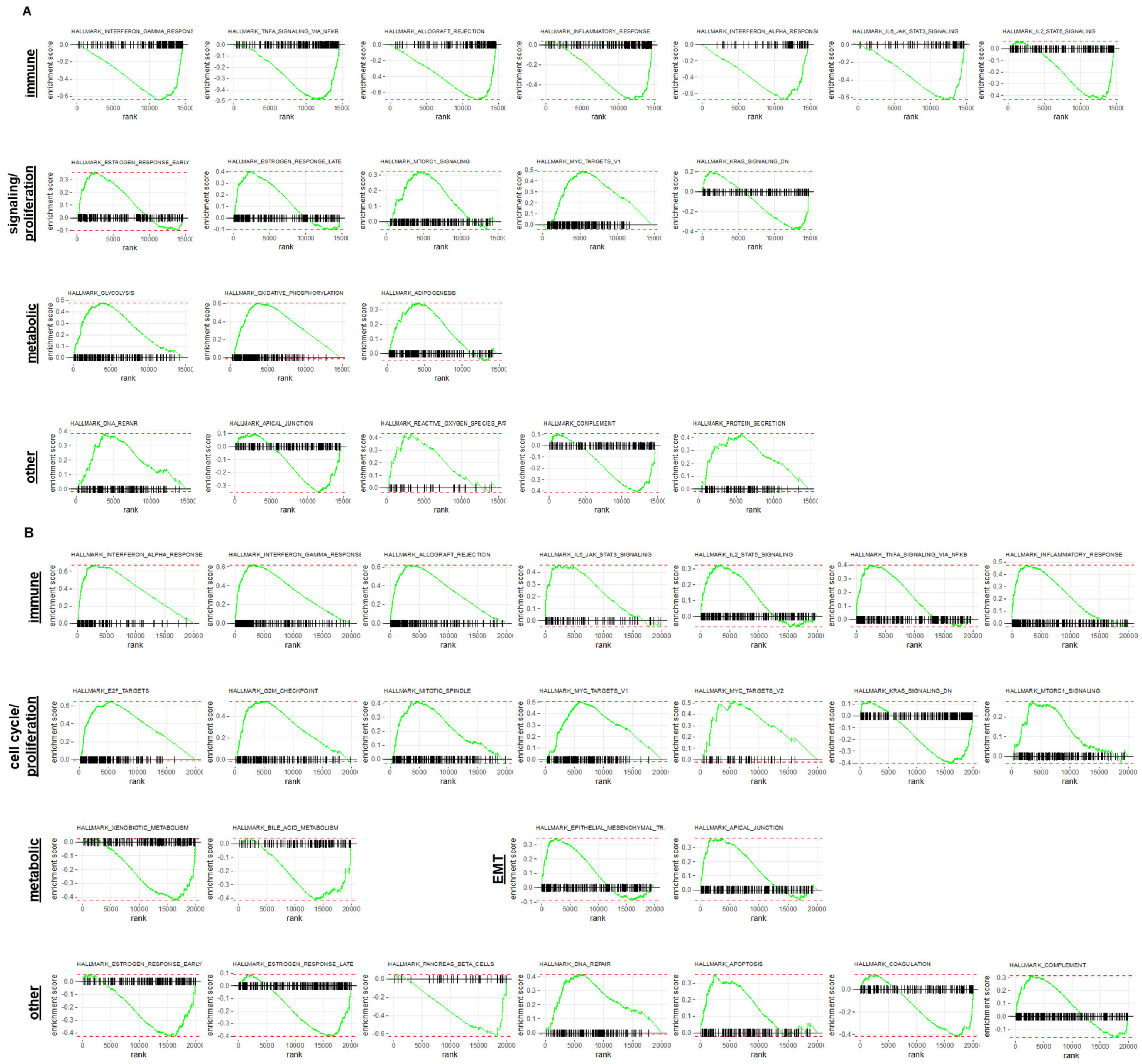

Supplemental Figure 7. GSEA of Hallmark pathways. A) GSEA enrichment score for the top 20 Hallmark pathways in matched primary LUAD versus BMs. **B)** GSEA enrichment score for the top 25 Hallmark pathways in *MET*-amplified versus non-*MET*-amplified BMs.

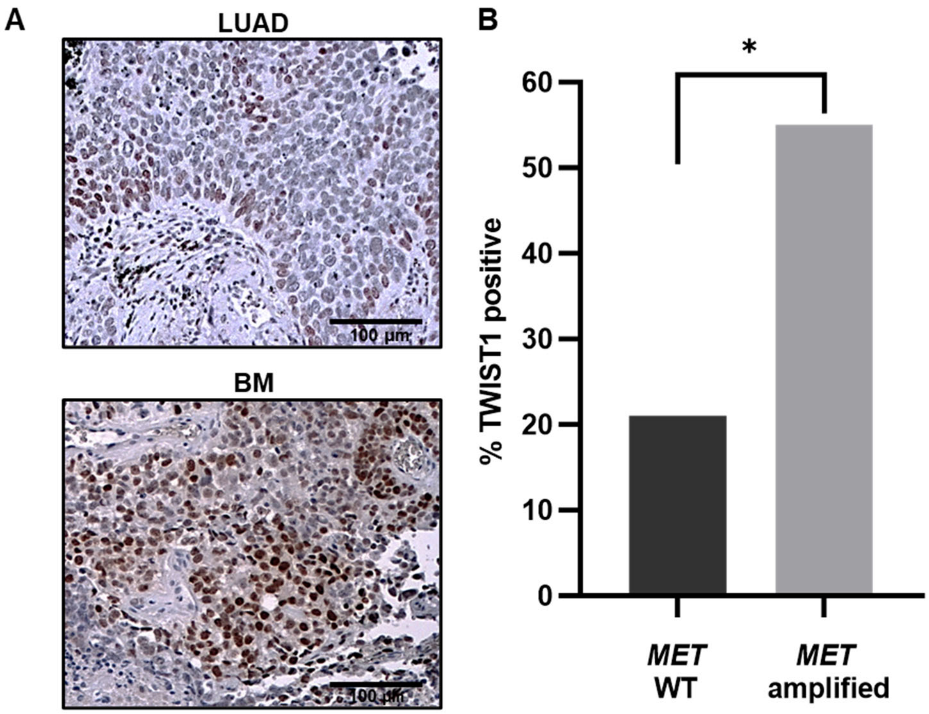

Supplemental Figure 8. TWIST1 is more frequently expressed in BM cases with *MET* amplification. TWIST1 IHC was performed on FFPE tissue from a subset of BM cases with available tissue (N=52). **A)** Representative TWIST1 IHC staining in a paired primary lung tumor (LUAD) and corresponding BM from the same patient. Scale bar= 100μm. **B)** Frequency of TWIST1 positivity in *MET* WT versus *MET*-amplified BM cases (P=0.047; Fisher's exact test, 2-sided).

A

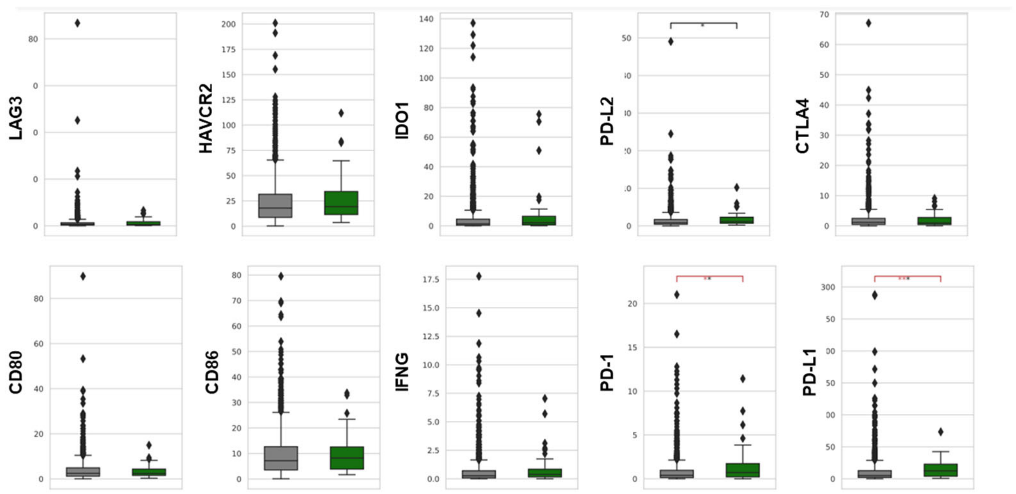

	Median *MET*WT	Median *MET*-altered	p-value	q-value
LAG3	0.664	0.588	0.903	0.903
HAVCR2	17.947	19.328	0.261	0.435
IDO1	1.389	2.133	0.175	0.350
PD-L2	0.824	1.146	***0.019***	***0.063***
CTLA4	1.162	0.933	0.730	0.811
CD80	2.438	2.371	0.621	0.776
CD86	7.176	8.198	0.407	0.582
IFNG	0.260	0.382	0.0828	0.207
PD-1	0.395	0.721	***0.007***	***0.037***
PD-L1	5.047	12.422	***0.0005***	***0.005***

B

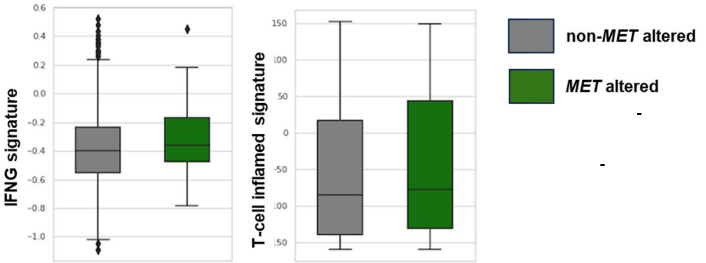

C

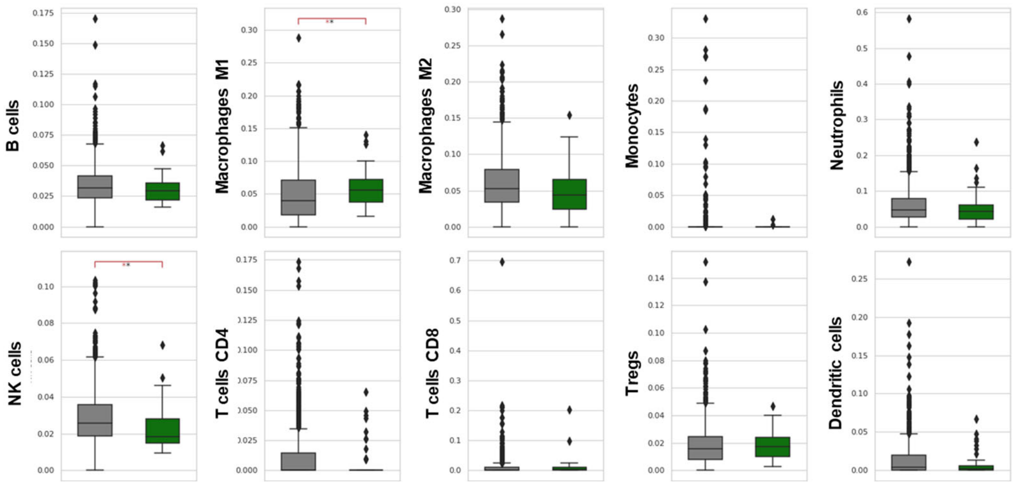

	Median *MET*WT	Median *MET*-altered	p-value	q-value
Dendritic cells	0.004	0.002	0.298	0.375
Macrophages M2	0.052	0.044	**0.082**	0.206
Macrophages M1	0.039	0.056	***0.006***	***0.028***
Monocytes	0.000	0.000	0.826	0.826
NK cells	0.026	0.018	***0.001***	***0.014***
T cells CD4	0.000	0.000	0.054	0.178
B cells	0.032	0.029	0.135	0.269
T cells CD8	0.002	0.004	0.216	0.359
Neutrophils	0.047	0.043	0.300	0.375
Tregs	0.016	0.017	0.398	0.442

Supplemental Figure 9. Quantiseq RNA deconvolution analysis in Caris dataset on non-*MET*-altered versus *MET*-altered BMs. A) Inhibitory checkpoint molecules, **B)** immune cell signatures, and **C)** phenotypes in non-*MET*-altered (gray) versus *MET*-altered (green) BMs. Tables show median values, p-value and q-value, Chi square test. Red text indicates significant differences between the 2 groups.

A

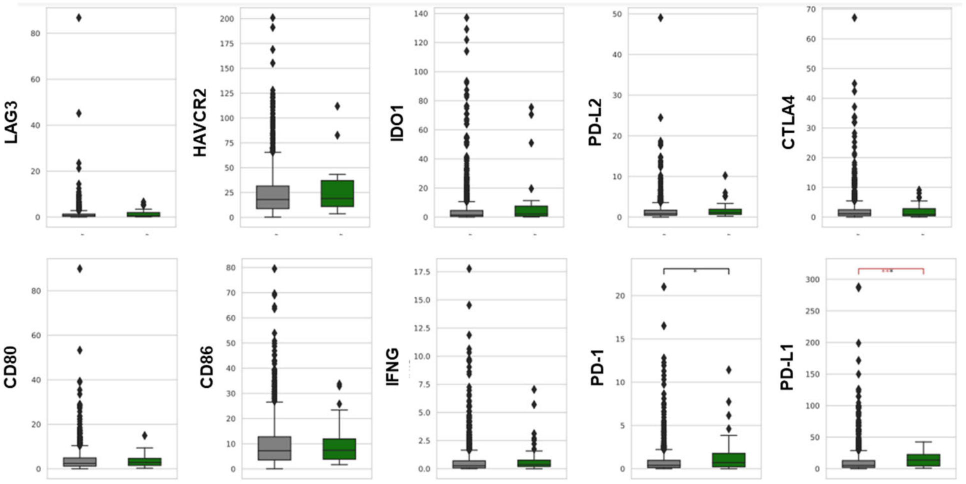

	Median *MET* WT	Median *MET*-amplified	p-value	q-value
LAG3	0.661	0.593	0.683	0.783
HAVCR2	17.975	18.999	0.375	0.626
IDO1	1.389	2.133	0.168	0.335
PD-L2	0.828	1.113	0.052	0.173
CTLA4	1.164	0.900	0.771	0.783
CD80	2.415	2.821	0.549	0.783
CD86	7.234	7.431	0.783	0.783
IFNG	0.262	0.355	0.074	0.185
PD-1	0.397	0.706	***0.013***	***0.065***
PD-L1	5.084	13.770	***0.001***	***0.010***

B

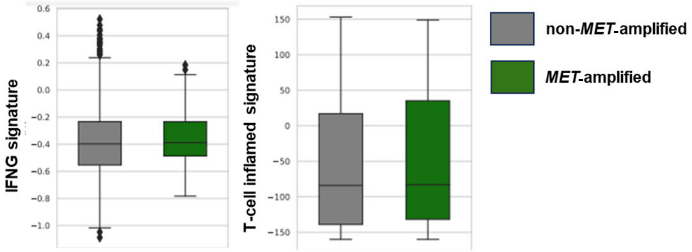

C

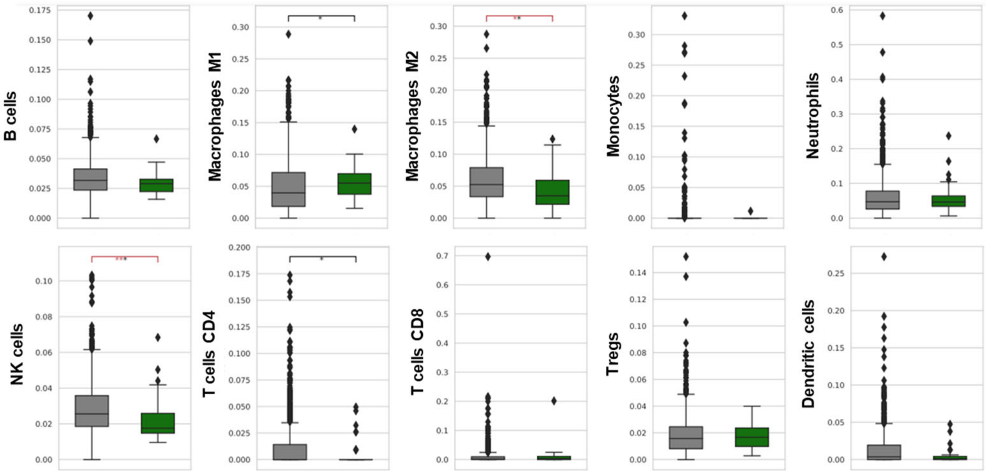

	Median *MET* WT	Median *MET*-amplified	p-value	q-value
Dendritic cells	0.004	0.001	0.093	0.154
Macrophages M2	0.052	0.035	***0.004***	***0.021***
Macrophages M1	0.040	0.055	***0.041***	0.102
Monocytes	0.000	0.000	0.529	0.720
NK cells	0.026	0.018	***0.001***	***0.006***
T cells CD4	0.000	0.000	***0.040***	0.102
B cells	0.032	0.029	0.073	0.145
T cells CD8	0.002	0.003	0.608	0.720
Neutrophils	0.047	0.046	0.891	0.891
Tregs	0.016	0.017	0.648	0.720

Supplemental Figure 10. Quantiseq RNA deconvolution analysis in Caris dataset of non-*MET*-amplified versus *MET*-amplified BMs. A) Inhibitory checkpoint molecules, **B)** immune cell signatures, and **C)** phenotypes in non-*MET*-amplified (gray) versus *MET*-amplified (green) BMs. Tables show median values, p-value and q-value, Chi-square test. Red text indicates significant differences between the 2 groups.

Supplemental Table 1. Selected patient characteristics for UPMC *MET* IHC cohort.

	***MET*-amplified brain metastases *N* = 36**	**non-*MET*-amplified brain metastases *N* = 13**	***P*-value**
**Age at diagnosis in yrs**			
mean (± sd)	59.4 (± 10.0)	62.2 (± 10.4)	n.s.
range	37-80	47-80	
**Sex, N (%)**			
Male	20 (55.6)	6 (46.2)	n.s.
Female	16 (44.4)	7 (53.8)	
**Race, N (%)**			
White	33 (91.7)	12 (92.3)	n.s.
Black/African American	3 (8.3)	1 (7.7)	
American Indian/Alaska Native	0 (0)	0 (0)	
Asian	0 (0)	0 (0)	
**Smoking status, N (%)**			
Ever	35 (97.2)	13 (100)	n.s.
Never	1 (2.8)	0 (0)	
**Metastases, N (%)**			
Synchronous	22 (61.1)	9 (69.2)	n.s
Metachronous	14 (38.9)	4 (30.8)	
**Alive at last follow up, N (%)**	2 (5.6)	1 (7.7)	n.s.

*Chi-square test used to determine *P*-value; N/A – not applicable

Supplemental Table 2. UPMC patient cohort with and without focal *MET* amplification in primary lung tumor.

	Focal *MET* amp positive (*N* = 85)	Focal *MET* amp negative (*N* = 131)	*P*-value*
Age at diagnosis in yrs.			
Average	64.8	67.0	n.s.
Range	42-87	41-87	
Sex, N (%)			
Female	49 (57.6%)	82 (62.6%)	n.s.
Male	36 (42.4%)	49 (37.4%)	
Smoking status, N (%)			
Ever	74 (87.1%)	110 (84.0%)	n.s.
Never	10 (11.8%)	19 (14.5%)	
Unknown	1 (1.1%)	2 (1.5%)	
Stage, N (%)			
I-II	13 (15.3%)	25 (19.1%)	n.s.
III	16 (18.8%)	34 (26.0%)	
IV	56 (65.9%)	72 (54.9%)	
Brain metastases, N (%)			
Yes	28 (33.0%)	49 (37%)	n.s.
No	57 (67.0%)	82 (63%)	
Brain metastases, N (%)			
Metachronous	11(39.3%)	20 (40.8%)	n.s.
Synchronous	17 (60.7%)	29 (59.2%)	

*Chi-square test used to determine *P*-value

Supplemental Table 3: Variants found in primary LUAD cases (*N* = 180).

Case Number	Hugo_Symbol	HGVSp_Short	Frequency (%)
LA159	ABL1	p.H415Y	0.56
LA29	ABL1	p.D400N	0.56
	ABL1	***Total***	***1.11***
LA177	AKT	p.E17K	0.56
LA161	AKT	p.E49K	0.56
	AKT	***Total***	***1.11***
LA159	ALK	p.D1203N	*0.56*
	ALK	***Total***	***0.56***
LA109, LA136, LA179, LA63, LA96	APC	p.E1317Q	2.78
LA176	APC	p.I1307K	0.56
LA176	APC	p.I1307Kfs*14	0.56
LA2	APC	p.P1433L	0.56
LA61	APC	p.P870S	0.56
LA159	APC	p.Q1095*	0.56
LA9	APC	p.R1103K	0.56
LA91	APC	p.T1292M	0.56
	APC	***Total***	***6.11***[1]
LA133, LA158, LA172, LA69	ATM	p.F858L	2.22
LA9	ATM	p.L2866F	0.56
LA183	ATM	p.S1691R	0.56
LA159	ATM	p.S2685L	0.56
LA159	ATM	p.W3052*	0.56
	ATM	***Total***	***3.89***
LA138	BRAF	p.G469A	0.56
LA65	BRAF	p.K601N	0.56
LA159	BRAF	p.Q461*	0.56
LA141	BRAF	p.V600E	0.56
	BRAF	***Total***	***2.22***
LA161	CDKN2A	p.A86G	0.56
LA110	CDKN2A	p.D108N	0.56
LA121	CDKN2A	p.G122D	0.56
LA73	CDKN2A	p.G67V	0.56
LA40	CDKN2A	p.G89C	0.56
LA4, LA52, LA67	CDKN2A	p.H83Y	1.67
LA131	CDKN2A	p.L117P	0.56
LA64	CDKN2A	p.R58*	0.56
LA152	CDKN2A	p.V82M	0.56
	CDKN2A	***Total***	***6.11***
LA105	CTNNB1	p.D32Y	*0.56*
LA147, LA64	CTNNB1	p.G34R	1.11
LA90	CTNNB1	p.S37F	*0.56*
	CTNNB1	***Total***	***2.22***
LA64	EGFR	p.A859T	0.56

LA110	EGFR	p.E734K	0.56
LA135, LA164, LA183	EGFR	p.E746_A750del	1.67
LA164, LA183	EGFR	p.E746_E749del	1.11
LA102	EGFR	p.G719A	0.56
LA159	EGFR	p.G719S	0.56
LA86	EGFR	p.L747_P753delinsS	0.56
LA89	EGFR	p.L747_T751del	0.56
LA35	EGFR	p.L747S	0.56
LA148, LA161, LA162, LA3, LA54, LA81, LA96	EGFR	p.L858R	3.89
LA36, LA56	EGFR	p.L861Q	1.11
LA63	EGFR	p.N771_P772insAH	0.56
LA9	EGFR	p.P772L	0.56
LA36	EGFR	p.R776H	0.56
LA35	EGFR	p.T751_A755del	0.56
LA96	EGFR	p.T790M	0.56
LA41	EGFR	p.V769_D770insTTP	0.56
	EGFR	***Total***	***12.22***
LA62	ERBB2	p.V777L	0.56
	ERBB2	***Total***	***0.56***
LA8	ERBB4	p.G586D	0.56
LA159	ERBB4	p.T926M	0.56
	ERBB4	***Total***	***1.11***
LA9	FBXW7	p.P274L	0.56
	FBXW7	***Total***	***0.56***
LA8	FGFR1	p.H284Y	0.56
	FGFR1	***Total***	***0.56***
LA58, LA83	FGFR3	p.F386L	1.11
LA159	FGFR3	p.R248C	0.56
	FGFR3	***Total***	***1.67***
LA9	GNAQ	p.D243N	0.56
	GNAQ	***Total***	***0.56***
LA50	GNAS	p.R844C	0.56
LA121	GNAS	p.R844H	0.56
	GNAS	***Total***	***1.11***
LA159	HNF1A	p.R200W	0.56
LA161	HNF1A	p.R203C	0.56
LA159	HNF1A	p.R263H	0.56
	HNF1A	***Total***	***1.11***
LA114	IDH1	p.R132G	0.56
LA126	IDH1	p.R132L	0.56
	IDH1	***Total***	***1.11***
LA64	IDH2	p.H173Y	0.56
LA109	IDH2	p.R140Q	0.56
	IDH2	***Total***	***1.11***
LA75, LA97	JAK3	p.P132T	1.11
LA119, LA166, LA17, LA23, LA31, LA45, LA59, LA81, LA83, LA84	JAK3	p.V722I	5.56

	JAK3	***Total***	***6.67***
LA159, LA9	KDR	p.A1218T	1.11
LA13	KDR	p.E1203*	0.56
LA9	KDR	p.T1350I	0.56
	KDR	***Total***	***1.67***
LA73	KRAS	p.A146V	0.56
LA13, LA4, LA8	KRAS	p.G12A	1.67
LA106, LA92, LA93, LA95, LA117, LA120, LA124, LA126, LA127, LA157, LA168, LA169, LA170, LA27, LA33, LA34, LA46, LA59, LA60, LA77	KRAS	p.G12C	11.11
LA105, LA9, LA50, LA38, LA142, LA70	KRAS	p.G12D	3.33
LA110	KRAS	p.G12S	0.56
LA100, LA157, LA151, LA154, LA52, LA6, LA82, LA11, LA114, LA132, LA137, LA147	KRAS	p.G12V	6.11
LA140, LA153	KRAS	p.G13C	1.11
LA136	KRAS	p.G13D	0.56
LA165	KRAS	p.Q61H	0.56
	KRAS	***Total***	***25.56***
LA76	MET	p.E168D	0.56
LA102, LA11, LA132, LA149, LA150, LA16, LA24, LA28, LA4	MET	p.N375S	5.00
LA9	MET	p.P822S	0.56
LA20, LA49	MET	p.R988C	1.11
LA101, LA133, LA143, LA53, LA6, LA63, LA90, LA129	MET	p.T1010I	4.44
LA9	MET	p.V1007I	0.56
LA48	MET	p.X981_splice	0.56
	MET	***Total***	***12.22***
LA13	NOTCH1	p.G2447C	0.56
LA64	NOTCH1	p.P2438S	0.56
LA9	NOTCH1	p.Q2440*	0.56
LA159	NOTCH1	p.R1598H	0.56
LA159	NOTCH1	p.S1588N	0.56
LA9	NOTCH1	p.T2466M	0.56
LA102, LA132, LA144, LA161, LA167, LA181, LA44, LA94	NOTCH1	p.V1578del	4.44
	NOTCH1	*Total*	**6.67**
LA159	NPM1	p.Q285*	0.56
LA159	NPM1	p.R291K	0.56
	NPM1	***Total***	***0.56***
LA76	NRAS	p.G13R	0.56
	NRAS	***Total***	***0.56***
LA159	PIK3CA	p.E1034G	0.56
LA127	PIK3CA	p.E542K	0.56
LA149, LA169, LA176, LA78	PIK3CA	p.E545K	2.22
LA159	PIK3CA	p.E547K	0.56
LA159	PIK3CA	p.H1047Y	0.56
LA102, LA83, LA84, LA9, LA97, LA99, LA121, LA123, LA140, LA148, LA151, LA156, LA159, LA16, LA161, LA169, LA170, LA178, LA22, LA25,	PIK3CA	p.I391M	17.78

LA28, LA29, LA36, LA42, LA46, LA55, LA60, LA61, LA62, LA67, LA72, LA75, LA78			
LA104	PIK3CA	p.K111N	0.56
LA131	PIK3CA	p.M1043I	0.56
	PIK3CA	***Total***	***21.11***
LA159	PTEN	p.D24N	0.56
LA176	PTEN	p.D252Y	0.56
LA133	PTEN	p.E314K	0.56
LA180	PTEN	p.K125E	0.56
LA159	PTEN	p.P248S	*0.56*
LA159	PTEN	p.S338F	0.56
LA9	PTEN	p.V133A	0.56
	PTEN	***Total***	***2.78***
LA9	PTPN11	p.V518I	0.56
	PTPN11	***Total***	***0.56***
LA105	RB1	p.D718G	0.56
LA2	RB1	p.I680T	0.56
	RB1	***Total***	***1.11***
LA30	SMAD4	p.E526K	0.56
LA159	SMAD4	p.G473E	0.56
LA75	SMAD4	p.Q183*	0.56
LA35	SMAD4	p.R361C	0.56
LA159	SMAD4	p.S343P	0.56
	SMAD4	***Total***	***2.22***
LA9	SMARCB1	p.D196Y	0.56
LA9	SMARCB1	p.W385*	0.56
	SMARCB1	***Total***	***0.56***
LA9	SMO	p.S308F	0.56
	SMO	***Total***	***0.56***
LA137	STK11	p.D53Tfs*11	0.56
LA1	STK11	p.E199*	0.56
LA4	STK11	p.E57Kfs*7	0.56
LA64	STK11	p.G171S	0.56
LA147	STK11	p.H168R	0.56
LA10	STK11	p.I274Sfs*13	0.56
LA5	STK11	p.L282Sfs*5	0.56
LA6	STK11	p.P281Rfs*6	0.56
LA128, LA48	STK11	p.X155_splice	1.11
	STK11	***Total***	***5.56***
LA13, LA5	TP53	p.A159P	1.11
LA9	TP53	p.C229Y	0.56
LA100	TP53	p.C242F	0.56
LA20	TP53	p.C275F	0.56
LA134	TP53	p.C277G	0.56
LA161	TP53	p.D184N	0.56
LA29	TP53	p.E171*	0.56
LA76	TP53	p.E198*	0.56

LA170, LA23, LA88	TP53	p.E204*	1.67
LA64	TP53	p.E221*	0.56
LA157	TP53	p.E271Q	0.56
LA157	TP53	p.E285K	0.56
LA48	TP53	p.E346*	0.56
LA106	TP53	p.G154V	0.56
LA64	TP53	p.G199V	0.56
LA51	TP53	p.G244C	0.56
LA110	TP53	p.G245C	0.56
LA142, LA94	TP53	p.G245V	1.11
LA64, LA9	TP53	p.G266V	1.11
LA83	TP53	p.G334V	0.56
LA89	TP53	p.H179R	0.56
LA64	TP53	p.H179Y	0.56
LA61	TP53	p.I195N	0.56
LA29	TP53	p.K132N	0.56
LA158	TP53	p.K164*	0.56
LA149	TP53	p.M237I	0.56
LA89	TP53	p.P72A	0.56
LA64, LA9	TP53	p.P98L	1.11
LA65	TP53	p.Q100*	0.56
LA80	TP53	p.Q104*	0.56
LA68, LA8	TP53	p.Q136E	1.11
LA131	TP53	p.R158L	0.56
LA12, LA7	TP53	p.R196*	1.11
LA2, LA80	TP53	p.R248L	1.11
LA136	TP53	p.R248Q	0.56
LA104, LA71	TP53	p.R249G	1.11
LA137	TP53	p.R249S	0.56
LA149	TP53	p.R267Q	0.56
LA115	TP53	p.R273C	0.56
LA154, LA180, LA3	TP53	p.R273L	1.67
LA52	TP53	p.R280G	0.56
LA31	TP53	p.R283H	0.56
LA133	TP53	p.S241F	0.56
LA161	TP53	p.S96F	0.56
LA69	TP53	p.T155P	0.56
LA153	TP53	p.T256A	0.56
LA10, LA7, LA84	TP53	p.V157F	1.67
LA64	TP53	p.V203L	0.56
LA174	TP53	p.V216M	0.56
LA140	TP53	p.V274A	0.56
LA116	TP53	p.V73Wfs*50	0.56
LA64	TP53	p.W91*	0.56
LA118, LA68	TP53	p.X187_splice	1.11
LA72	TP53	p.X307_splice	0.56

LA33	TP53	p.Y205F	0.56
LA176	TP53	p.Y220C	0.56
LA11, LA155	TP53	p.Y236C	1.11
	TP53	***Total***	***31.67***

[1] Total % Frequency is defined as total number of cases with one or more variant in a particular gene divided total number of cases.

Supplemental Table 4: Variants found in all LUAD BM cases (*N* = 74).

Case Number	Hugo_Symbol	HGVSp_Short	Frequency (%)
BA39,BA48	ABL1	p.G278V	2.70
BA38	ABL1	p.C349Y	1.35
BA35	ABL1	p.S429T	1.35
BA17	ABL1	p.Y372*	1.35
	ABL1	***Total***	***6.76***
BA17,BA22,BA39,BA42,BA48	ALK	p.G1184E	6.76
BA39,BA42	ALK	p.K1173I	2.70
	ALK	***Total***	***6.76***[1]
BA17,BA22,BA23,BA27,BA38,BA39,BA42,BA48, BA5,BA56,BA57,BA59,BA63,BA73	APC	p.P1433L	18.92
BA39,BA48	APC	p.A888T	2.70
BA26	APC	p.A1582P	1.35
BA61	APC	p.E1317Q	1.35
BA57	APC	p.N1122H	1.35
BA18	APC	p.N1548Kfs*11	1.35
BA48	APC	p.Q1095R	1.35
	APC	***Total***	***22.97***
BA42,BA56,BA58	ATM	p.F858L	4.05
BA47	ATM	p.D2448H	1.35
BA42	ATM	p.L2885P	1.35
BA22	ATM	p.Q2730*	1.35
BA62	ATM	p.Q852*	1.35
BA60	ATM	p.S1691R	1.35
	ATM	***Total***	***9.46***
BA33	CDH1	p.G78D	1.35
	CDH1	***Total***	***1.35***
BA37	CDKN2A	p.A68T	1.35
BA45	CDKN2A	p.D108V	1.35
BA14	CDKN2A	p.E61*	1.35
BA31	CDKN2A	p.E69Qfs*49	1.35
BA4	CDKN2A	p.H83Y	1.35
BA46	CDKN2A	p.R58*	1.35
BA48	CDKN2A	p.R80*	1.35
BA17	CDKN2A	p.W110*	1.35
	CDKN2A	***Total***	***10.81***
BA17,BA27,BA3	EGFR	p.L858R	4.05
BA38,BA39,BA42,BA48	EGFR	p.T783A	5.41
BA38,BA42,BA48	EGFR	p.A822T	4.05
BA38,BA62	EGFR	p.E746_A750del	2.70
BA62	EGFR	p.E746_E749del	1.35
BA38	EGFR	p.G810S	1.35
BA55	EGFR	p.S768I	1.35
	EGFR	***Total***	***12.16***
BA25	ERBB2	p.Y772_A775dup	1.35

	ERBB2	***Total***	***1.35***
BA39,BA48	FBXW7	p.T482A	2.70
	FBXW7	***Total***	***2.70***
BA39,BA42,BA48	FGFR3	p.N646D	4.05
BA18,BA56	FGFR3	p.F386L	2.70
BA36	FGFR3	p.A719T	1.35
BA25	FGFR3	p.G377D	1.35
	FGFR3	***Total***	***9.46***
BA39,BA48	*FLT3*	p.K602R	2.70
BA51	FLT3	p.G583D	1.35
BA39	FLT3	p.S684P	1.35
	FLT3	***Total***	***4.05***
BA48	GNA11	p.G208C	1.35
	GNA11	***Total***	***1.35***
BA39,BA48	IDH1	p.L120P	2.70
BA39,BA42	IDH1	p.P118L	2.70
	IDH1	***Total***	***4.05***
BA48	IDH2	p.K155N	1.35
BA38	IDH2	p.W164*	1.35
	IDH2	***Total***	***2.70***
BA18,BA35,BA72	JAK3	p.V722I	4.05
BA17	JAK3	p.T714A	1.35
	JAK3	***Total***	***5.41***
BA13	KDR	p.E1203*	1.35
BA39	KDR	p.G1348E	1.35
BA62	KDR	p.R962H	1.35
BA17	KDR	p.T1350I	1.35
	KDR	***Total***	***5.41***
BA11,BA30,BA34,BA35,BA40,BA46,BA50,BA52, BA55,BA60,BA67, BA68	KRAS	p.G12C	16.22
BA15,BA33,BA47,BA6,BA66,BA74	KRAS	p.G12V	8.11
BA16,BA24,BA53,BA64,BA9	KRAS	p.G12D	6.76
BA13,BA4,BA8	KRAS	p.G12A	4.05
BA48,BA49,BA58	KRAS	p.Q61H	4.05
BA71,BA72	KRAS	p.G13C	2.70
BA19,BA20	KRAS	p.G13D	2.70
BA23	KRAS	p.Q61K	1.35
BA65	KRAS	p.Q61L	1.35
BA73	KRAS	p.Q61R	1.35
	KRAS	***Total***	***48.65***
BA11,BA20,BA21,BA4,BA43,BA44	MET	p.N375S	8.11
BA38,BA58,BA59,BA6,BA70,BA74	MET	p.T1010I	8.11
BA39,BA42,BA48	MET	p.A179T	4.05
BA29	MET	p.H1112Y	1.35
BA38	MET	p.H855Y	1.35
	MET	***Total***	***21.62***
BA39	MLH1	p.Q391H	1.35

BA71	MLH1	p.R389Q	1.35
BA71	MLH1	p.V384D	1.35
	MLH1	***Total***	***2.70***
BA22,BA42	NOTCH1	p.H1601L	2.70
BA74	NOTCH1	p.E1583D	1.35
BA13	NOTCH1	p.G2447C	1.35
BA74	NOTCH1	p.M1580L	1.35
BA41	NOTCH1	p.R1568G	1.35
BA74	NOTCH1	p.R1598H	1.35
BA74	NOTCH1	p.S1588N	1.35
	NOTCH1	***Total***	***6.76***
BA39	NRAS	p.G12V	1.35
	NRAS	***Total***	***1.35***
BA62	PDGFRA	p.P653S	1.35
	PDGFRA	***Total***	***1.35***
BA19,BA20,BA24,BA33,BA36,BA38,BA41,BA44, BA47,BA49,BA51,BA57,BA65,BA73,BA9	PIK3CA	p.I391M	20.27
BA38,BA57	PIK3CA	p.E542K	2.70
BA41	PIK3CA	p.E417Q	1.35
BA74	PIK3CA	p.M1040I	1.35
BA54	PIK3CA	p.V344M	1.35
	PIK3CA	***Total***	***22.97***
BA38	PTEN	p.K267Rfs*9	1.35
BA56	PTEN	p.N63Kfs*11	1.35
	PTEN	***Total***	***2.70***
BA26	PTPN11	p.G503V	1.35
	PTPN11	***Total***	***1.35***
BA17,BA22,BA23,BA37,BA38,BA39,BA42,BA48, BA53,BA56,BA71,BA73	RB1	p.I680T	16.22
BA39,BA42,BA48	RB1	p.L670P	4.05
BA24,BA30,BA55	RB1	p.T766Nfs*29	4.05
BA71	RB1	p.Q575Tfs*10	1.35
	RB1	***Total***	***20.27***
BA22,BA23,BA39,BA42,BA48,BA56,BA59,BA73	RET	p.E884V	10.81
BA39	RET	p.D892Y	1.35
BA17	RET	p.E901K	1.35
BA48	RET	p.M918V	1.35
	RET	***Total***	***12.16***
BA17,BA22,BA23,BA27,BA38,BA39,BA42,BA48, BA53,BA56,BA73,BA9	SMAD4	p.V354L	16.22
BA17,BA22,BA23,BA39,BA42,BA48,BA53,BA56, BA59	SMAD4	p.Q256L	12.16
BA54	SMAD4	p.C363S	1.35
BA40	SMAD4	p.C499R	1.35
BA48	SMAD4	p.I347V	1.35
BA49	SMAD4	p.R189C	1.35
BA33	SMAD4	p.R361C	1.35
	SMAD4	***Total***	***22.97***

BA48	SMARCB1	p.E59*	1.35
BA22	SMARCB1	p.I63Nfs*8	1.35
	SMARCB1	***Total***	***2.70***
BA47	SMO	p.A406S	1.35
	SMO	***Total***	***1.35***
BA22,BA66	STK11	p.D194Y	2.70
BA47,BA60	STK11	p.X155_splice	2.70
BA58	STK11	p.E199*	1.35
BA4	STK11	p.E57Kfs*7	1.35
BA10	STK11	p.I274Sfs*13	1.35
BA5	STK11	p.L282Sfs*5	1.35
BA6	STK11	p.P281Rfs*6	1.35
BA32	STK11	p.R40Sfs*6	1.35
	STK11	***Total***	***13.51***
BA10,BA24,BA30,BA39,BA7	TP53	p.V157F	6.76
BA21,BA33,BA61,BA72	TP53	p.R158L	5.41
BA3,BA38,BA42,BA70	TP53	p.R273L	5.41
BA18,BA26,BA5	TP53	p.A159P	4.05
BA23,BA3	TP53	p.E286V	2.70
BA72,BA9	TP53	p.G266V	2.70
BA12,BA7	TP53	p.R196*	2.70
BA2,BA47	TP53	p.R248L	2.70
BA58,BA65	TP53	p.R248Q	2.70
BA22,BA42	TP53	p.S96P	2.70
BA39,BA48	TP53	p.V97A	2.70
BA40,BA74	TP53	p.Y220C	2.70
BA56	TP53	p.A161T	1.35
BA71	TP53	p.C176F	1.35
BA15	TP53	p.C176S	1.35
BA45	TP53	p.C242F	1.35
BA35	TP53	p.C275Lfs*31	1.35
BA57	TP53	p.E198*	1.35
BA22	TP53	p.E285K	1.35
BA29	TP53	p.E294*	1.35
BA48	TP53	p.E298*	1.35
BA49	TP53	p.G154V	1.35
BA1	TP53	p.G226Wfs*3	1.35
BA56	TP53	p.G302Rfs*4	1.35
BA36	TP53	p.G334V	1.35
BA30	TP53	p.H179Y	1.35
BA46	TP53	p.H193P	1.35
BA67	TP53	p.H193R	1.35
BA64	TP53	p.K351*	1.35
BA54	TP53	p.N247I	1.35
BA68	TP53	p.P151H	1.35
BA68	TP53	p.P152Afs*28	1.35

BA63	TP53	p.P82Rfs*41	1.35
BA22	TP53	p.P92S	1.35
BA8	TP53	p.Q136E	1.35
BA69	TP53	p.Q167*	1.35
BA73	TP53	p.R110L	1.35
BA16	TP53	p.R174W	1.35
BA32	TP53	p.R175H	1.35
BA34	TP53	p.R202L	1.35
BA33	TP53	p.R213*	1.35
BA38	TP53	p.R282W	1.35
BA28	TP53	p.R333Lfs*12	1.35
BA14	TP53	p.R342*	1.35
BA44	TP53	p.S215I	1.35
BA34	TP53	p.V203L	1.35
BA25	TP53	p.V216L	1.35
BA50	TP53	p.W91Cfs*30	1.35
BA43	TP53	p.X307_splice	1.35
	TP53	***Total***	***74.32***
BA38,BA39,BA42,BA48	VHL	p.V142G	5.41
BA74	VHL	p.V87L	1.35
	VHL	***Total***	***6.76***

[1] Total % Frequency is defined as total number of cases with one or more variant in a particular gene divided total number of cases.

Supplemental Table 5: *MET* variants in Caris cohort.

Coding DNA sequence change	Amino Acid Mutation
c.2942-10_2958del27	MET exon 14 skipping mutation
c.2942-102_2942-5del98	MET exon 14 skipping mutation
c.2942-11_2942-1del11	MET exon 14 skipping mutation
c.2942-11_2963del33	MET exon 14 skipping mutation
c.2942-11_2967del37	MET exon 14 skipping mutation
c.2942-12_2945del16	MET exon 14 skipping mutation
c.2942-12_2976del47	MET exon 14 skipping mutation
c.2942-13_2942-2del12	MET exon 14 skipping mutation
c.2942-13_2945del17	MET exon 14 skipping mutation
c.2942-14_2942-2del13	MET exon 14 skipping mutation
c.2942-14_2942-3del12	MET exon 14 skipping mutation
c.2942-14_2942-4del11	MET exon 14 skipping mutation
c.2942-14_2945del18	MET exon 14 skipping mutation
c.2942-15_2942-2del14	MET exon 14 skipping mutation
c.2942-15_2942-3del13	MET exon 14 skipping mutation
c.2942-15_2942-4del12	MET exon 14 skipping mutation
c.2942-15_2945del19	MET exon 14 skipping mutation
c.2942-15_2952del26	MET exon 14 skipping mutation
c.2942-15_2957del31	MET exon 14 skipping mutation
c.2942-15_2958del32	MET exon 14 skipping mutation
c.2942-15_2985del59	MET exon 14 skipping mutation
c.2942-16_2942-3del14	MET exon 14 skipping mutation
c.2942-16_2942-4del13	MET exon 14 skipping mutation
c.2942-16_2943del18	MET exon 14 skipping mutation
c.2942-16_2945del20	MET exon 14 skipping mutation
c.2942-16_2947del22	MET exon 14 skipping mutation
c.2942-16_2957del32	MET exon 14 skipping mutation
c.2942-17_2942-1del17	MET exon 14 skipping mutation
c.2942-17_2942-3del15	MET exon 14 skipping mutation
c.2942-17_2942-4del14	MET exon 14 skipping mutation
c.2942-17_2942-9del9	MET exon 14 skipping mutation
c.2942-17_2945del21	MET exon 14 skipping mutation
c.2942-18_2942-1del18	MET exon 14 skipping mutation
c.2942-18_2942-2del17	MET exon 14 skipping mutation
c.2942-18_2942-3del16	MET exon 14 skipping mutation
c.2942-18_2942-4del15	MET exon 14 skipping mutation
c.2942-18_2942-5del14	MET exon 14 skipping mutation
c.2942-18_2942-6del13	MET exon 14 skipping mutation
c.2942-18_2942-7del12	MET exon 14 skipping mutation
c.2942-18_2942-8del11	MET exon 14 skipping mutation
c.2942-18_2942-9del10	MET exon 14 skipping mutation

c.2942-18_2942del19	MET exon 14 skipping mutation
c.2942-18_2945del22	MET exon 14 skipping mutation
c.2942-18_2956del33	MET exon 14 skipping mutation
c.2942-19_2942-11del9	MET exon 14 skipping mutation
c.2942-19_2942-1del19	MET exon 14 skipping mutation
c.2942-19_2942-2del18	MET exon 14 skipping mutation
c.2942-19_2942-3del17	MET exon 14 skipping mutation
c.2942-19_2942-4del16	MET exon 14 skipping mutation
c.2942-19_2942-9del11	MET exon 14 skipping mutation
c.2942-19_2946del24	MET exon 14 skipping mutation
c.2942-19_2947del25	MET exon 14 skipping mutation
c.2942-19_2953del31	MET exon 14 skipping mutation
c.2942-19_2957del35	MET exon 14 skipping mutation
c.2942-1G>A	MET exon 14 skipping mutation
c.2942-1G>C	MET exon 14 skipping mutation
c.2942-1G>T	MET exon 14 skipping mutation
c.2942-2_2966del27	MET exon 14 skipping mutation
c.2942-20_2942-11del10	MET exon 14 skipping mutation
c.2942-20_2942-1del20	MET exon 14 skipping mutation
c.2942-20_2942-2del19	MET exon 14 skipping mutation
c.2942-20_2942-3del18	MET exon 14 skipping mutation
c.2942-20_2942-4del17	MET exon 14 skipping mutation
c.2942-20_2942-9del12	MET exon 14 skipping mutation
c.2942-20_2945del24	MET exon 14 skipping mutation
c.2942-20_2947del26	MET exon 14 skipping mutation
c.2942-20_2953del32	MET exon 14 skipping mutation
c.2942-21_2942-1del21	MET exon 14 skipping mutation
c.2942-21_2942-3del19	MET exon 14 skipping mutation
c.2942-21_2942-9del13	MET exon 14 skipping mutation
c.2942-21_2943del23	MET exon 14 skipping mutation
c.2942-21_2945del25	MET exon 14 skipping mutation
c.2942-21_2957del37	MET exon 14 skipping mutation
c.2942-22_2942-10del13	MET exon 14 skipping mutation
c.2942-22_2942-2del21	MET exon 14 skipping mutation
c.2942-22_2942-3del20	MET exon 14 skipping mutation
c.2942-22_2942-5del18	MET exon 14 skipping mutation
c.2942-22_2942-7del16	MET exon 14 skipping mutation
c.2942-22_2950del31	MET exon 14 skipping mutation
c.2942-22_2962del43	MET exon 14 skipping mutation
c.2942-23_2942-2del22	MET exon 14 skipping mutation
c.2942-23_2947del29	MET exon 14 skipping mutation
c.2942-24_2942-1del24	MET exon 14 skipping mutation
c.2942-24_2946del29	MET exon 14 skipping mutation
c.2942-25_2942-4del22	MET exon 14 skipping mutation

c.2942-25_2942-5del21	MET exon 14 skipping mutation
c.2942-26_2942-11del16	MET exon 14 skipping mutation
c.2942-26_2942-2del25	MET exon 14 skipping mutation
c.2942-26_2942-3del24	MET exon 14 skipping mutation
c.2942-26_2942-6del21	MET exon 14 skipping mutation
c.2942-26_2946del31	MET exon 14 skipping mutation
c.2942-26_2950del35	MET exon 14 skipping mutation
c.2942-26_2958del43	MET exon 14 skipping mutation
c.2942-27_2942-10del18	MET exon 14 skipping mutation
c.2942-27_2942-12del16	MET exon 14 skipping mutation
c.2942-27_2942-14del14	MET exon 14 skipping mutation
c.2942-27_2942-7del21	MET exon 14 skipping mutation
c.2942-27_2942del28	MET exon 14 skipping mutation
c.2942-27_2944del30	MET exon 14 skipping mutation
c.2942-28_2942-10del19	MET exon 14 skipping mutation
c.2942-28_2942-2del27	MET exon 14 skipping mutation
c.2942-28_2942-4del25	MET exon 14 skipping mutation
c.2942-28_2942-8del21	MET exon 14 skipping mutation
c.2942-28_2942del29	MET exon 14 skipping mutation
c.2942-28_2944del31	MET exon 14 skipping mutation
c.2942-28_2950del37	MET exon 14 skipping mutation
c.2942-28_2955del42	MET exon 14 skipping mutation
c.2942-28_2969del56	MET exon 14 skipping mutation
c.2942-28_2977del64	MET exon 14 skipping mutation
c.2942-29_2942del30	MET exon 14 skipping mutation
c.2942-29_2950del38	MET exon 14 skipping mutation
c.2942-29_2974del62	MET exon 14 skipping mutation
c.2942-29_3013del101	MET exon 14 skipping mutation
c.2942-2A>C	MET exon 14 skipping mutation
c.2942-30_2942-11del20	MET exon 14 skipping mutation
c.2942-30_2942-12del19	MET exon 14 skipping mutation
c.2942-30_2942-14del17	MET exon 14 skipping mutation
c.2942-30_2942-8del23	MET exon 14 skipping mutation
c.2942-30_2945del34	MET exon 14 skipping mutation
c.2942-31_2942-9del23	MET exon 14 skipping mutation
c.2942-32_2942-11del22	MET exon 14 skipping mutation
c.2942-32_2943del34	MET exon 14 skipping mutation
c.2942-32_2945del36	MET exon 14 skipping mutation
c.2942-33_2945del37	MET exon 14 skipping mutation
c.2942-33_2949del41	MET exon 14 skipping mutation
c.2942-34_2942-10del25	MET exon 14 skipping mutation
c.2942-34_2942-7del28	MET exon 14 skipping mutation
c.2942-34_2946del39	MET exon 14 skipping mutation
c.2942-36_2942-14del23	MET exon 14 skipping mutation

c.2942-36_2942-8del29	MET exon 14 skipping mutation
c.2942-36_2944del39	MET exon 14 skipping mutation
c.2942-36_2964del59	MET exon 14 skipping mutation
c.2942-38_2942-1del38	MET exon 14 skipping mutation
c.2942-3A>G	MET exon 14 skipping mutation
c.2942-4_2961del24	MET exon 14 skipping mutation
c.2942-40_2943del42	MET exon 14 skipping mutation
c.2942-41_2942-9del33	MET exon 14 skipping mutation
c.2942-42_2942-15del28	MET exon 14 skipping mutation
c.2942-42_2942-2del41	MET exon 14 skipping mutation
c.2942-42_2946del47	MET exon 14 skipping mutation
c.2942-45_2946del50	MET exon 14 skipping mutation
c.2942-4T>A	MET exon 14 skipping mutation
c.2942-5_2961del25	MET exon 14 skipping mutation
c.2942-5_2970del34	MET exon 14 skipping mutation
c.2942-5_2978del42	MET exon 14 skipping mutation
c.2942-51_2942-7del45	MET exon 14 skipping mutation
c.2942-53_2989del101	MET exon 14 skipping mutation
c.2942-54_2944del57	MET exon 14 skipping mutation
c.2942-55_2945del59	MET exon 14 skipping mutation
c.2942-57_2942-7del51	MET exon 14 skipping mutation
c.2942-58_2946del63	MET exon 14 skipping mutation
c.2942-6_2942-2delTTTAA	MET exon 14 skipping mutation
c.2942-6_2961del26	MET exon 14 skipping mutation
c.2942-62_2942-12del51	MET exon 14 skipping mutation
c.2942-63_2942-9del55	MET exon 14 skipping mutation
c.2942-7_2942-2delTTTTAA	MET exon 14 skipping mutation
c.2942-7_2958del24	MET exon 14 skipping mutation
c.2942-7_2963del29	MET exon 14 skipping mutation
c.2942-7_2968del34	MET exon 14 skipping mutation
c.2942-8_2965del32	MET exon 14 skipping mutation
c.2942-8_2968del35	MET exon 14 skipping mutation
c.2946_2979del34	G983fs
c.2948_3029del82	G983fs
c.2959delins17	V987fs
c.2961dupT	R988fs
c.2962_2972del11	R988fs
c.2962_2973delinsT	R988fs
c.2962_2975del14	R988fs
c.2962_3014delinsA	R988fs
c.3048_3082+24del59	MET exon 14 skipping mutation
c.3049_3061delinsC	E1017_Y1021delinsH
c.3049_3082+2del36	MET exon 14 skipping mutation
c.3051_3082+24del56	MET exon 14 skipping mutation

c.3053_3073del21	S1018_T1024del
c.3057_3074del	D1020_F1025del
c.3058_3082+34del59	MET exon 14 skipping mutation
c.3058_3082+42del67	MET exon 14 skipping mutation
c.3058G>A	D1020N
c.3059_3082+11del35	MET exon 14 skipping mutation
c.3060_3082+26del49	MET exon 14 skipping mutation
c.3061 T > C	Y1021H
c.3061_3063delTAC	Y1021D
c.3061_3063delTAC	Y1021del
c.3061T>A	Y1021N
c.3061T>G	Y1021D
c.3062_3082+7del28	MET exon 14 skipping mutation
c.3062A>C	Y1021S
c.3062A>G	Y1021C
c.3062A>T	Y1021F
c.3063_3064delCC	Y1021fs
c.3063_3081del19	Y1021fs
c.3063_3082+3del23	MET exon 14 skipping mutation
c.3063_3082+5del25	MET exon 14 skipping mutation
c.3063_3082+6del26	MET exon 14 skipping mutation
c.3063_3082del20	Y1021fs
c.3064_3082+1del20	MET exon 14 skipping mutation
c.3065_3082+1del19	MET exon 14 skipping mutation
c.3066_3082+12del29	MET exon 14 skipping mutation
c.3067_3082+3del19	MET exon 14 skipping mutation
c.3068_3082+1del16	MET exon 14 skipping mutation
c.3069_3082+2del16	MET exon 14 skipping mutation
c.3069_3082del	A1023fs
c.3071_3082+10del22	MET exon 14 skipping mutation
c.3071_3082+15del27	MET exon 14 skipping mutation
c.3071_3082+1del13	MET exon 14 skipping mutation
c.3071_3082+3del15	MET exon 14 skipping mutation
c.3071_3082+4del16	MET exon 14 skipping mutation
c.3071_3082+5del17	MET exon 14 skipping mutation
c.3072_3082+8del19	MET exon 14 skipping mutation
c.3072_3082+9del20	MET exon 14 skipping mutation
c.3073_3082+14del24	MET exon 14 skipping mutation
c.3073_3082+2del12	MET exon 14 skipping mutation
c.3073_3082+4del14	MET exon 14 skipping mutation
c.3073_3082+8del18	MET exon 14 skipping mutation
c.3074_3082+14del23	MET exon 14 skipping mutation
c.3074_3082+17del26	MET exon 14 skipping mutation
c.3074_3082+4delTTCCAGAAGGTATinsG	F1025fs

c.3074_3082+9del18	MET exon 14 skipping mutation
c.3075_3082+2del10	MET exon 14 skipping mutation
c.3075_3082+4del12	MET exon 14 skipping mutation
c.3075_3082+8del16	MET exon 14 skipping mutation
c.3075_3082+9del17	MET exon 14 skipping mutation
c.3075_3082delinsC	P1026fs
c.3076_3080delCCAGA	P1026fs
c.3076_3081delinsT	P1026fs
c.3076_3082+13del20	MET exon 14 skipping mutation
c.3076_3082+16del23	MET exon 14 skipping mutation
c.3076_3082+19del26	MET exon 14 skipping mutation
c.3076_3082+4del11	MET exon 14 skipping mutation
c.3076_3082+7del14	MET exon 14 skipping mutation
c.3076_3082del7	P1026fs
c.3077_3081delCAGAA	P1026fs
c.3077_3082+1del7	MET exon 14 skipping mutation
c.3077_3082+9del15	MET exon 14 skipping mutation
c.3077_3082delCAGAAG	P1026_D1028delinsH
c.3078_3082+13del18	MET exon 14 skipping mutation
c.3078_3082+24del29	MET exon 14 skipping mutation
c.3078_3082+6del11	MET exon 14 skipping mutation
c.3079_3082+15del19	MET exon 14 skipping mutation
c.3079_3082+1delGAAGG	MET exon 14 skipping mutation
c.3079_3082+3del7	MET exon 14 skipping mutation
c.3079_3082+4del8	MET exon 14 skipping mutation
c.3079_3082+5del9	MET exon 14 skipping mutation
c.3080_3082+11del14	MET exon 14 skipping mutation
c.3080_3082+18del21	MET exon 14 skipping mutation
c.3080_3082+1delAAGG	MET exon 14 skipping mutation
c.3081_3082 + 2delAGGT	E1027fs
c.3081_3082+15del17	MET exon 14 skipping mutation
c.3081_3082+1delAGG	MET exon 14 skipping mutation
c.3081_3082+5del7	MET exon 14 skipping mutation
c.3082_3082+11del12	MET exon 14 skipping mutation
c.3082_3082+16del17	MET exon 14 skipping mutation
c.3082_3082+26del27	MET exon 14 skipping mutation
c.3082_3082+27del28	MET exon 14 skipping mutation
c.3082_3082+2delGGT	MET exon 14 skipping mutation
c.3082_3082+34del35	MET exon 14 skipping mutation
c.3082_3082+3delGGTA	MET exon 14 skipping mutation
c.3082_3082+5delGGTATA	MET exon 14 skipping mutation
c.3082_3082+7del8	MET exon 14 skipping mutation
c.3082+1_3082+11del11	MET exon 14 skipping mutation
c.3082+1_3082+13del13	MET exon 14 skipping mutation

c.3082+1_3082+2insA	MET exon 14 skipping mutation
c.3082+1_3082+3delGTA	MET exon 14 skipping mutation
c.3082+1delG	MET exon 14 skipping mutation
c.3082+1dupG	MET exon 14 skipping mutation
c.3082+1G>A	MET exon 14 skipping mutation
c.3082+1G>C	MET exon 14 skipping mutation
c.3082+1G>T	MET exon 14 skipping mutation
c.3082+2_3082+4delTAT	MET exon 14 skipping mutation
c.3082+2delT	MET exon 14 skipping mutation
c.3082+2T>A	MET exon 14 skipping mutation
c.3082+2T>C	MET exon 14 skipping mutation
c.3082+2T>G	MET exon 14 skipping mutation
c.3082+3_3082+12del10	MET exon 14 skipping mutation
c.3082+3A>C	MET exon 14 skipping mutation
c.3082+3A>G	MET exon 14 skipping mutation
c.3082+3A>T	MET exon 14 skipping mutation
c.3082+3delA	MET exon 14 skipping mutation
c.3082delinsCCTT	D1028delinsPY
c.3082G>A	D1028N
c.3082G>C	D1028H
c.3082G>T	D1028Y
c.3280C>T	H1112Y
c.3281A>G	H1112R
c.3334C>G	H1112D
c.3583C>G	L1213V
c.3736G>A	D1246N
c.3736G>C	D1246H
c.3742T>C	Y1248H
c.3743A>G	Y1248C
c.3803T>C	M1268T

Supplemental Table 6: Variants found in *MET* WT LUAD BM cases (*N* = 43).

Case Number	Hugo_Symbol	HGVSp_Short	Frequency (%)
BA17	ABL1	p.Y372*	2.33
	ABL1	***Total***	***2.33***
BA17, BA22	ALK	p.G1184E	4.65
	ALK	***Total***	***4.65***
BA17, BA22, BA23, BA27, BA5, BA56, BA57, BA63	APC	p.P1433L	18.60
BA26	APC	p.A1582P	2.33
BA57	APC	p.N1122H	2.33
BA18	APC	p.N1548Kfs*11	2.33
	APC	***Total***	***23.26***[1]
BA56	ATM	p.F858L	2.33
BA47	ATM	p.D2448H	2.33
BA22	ATM	p.Q2730*	2.33
BA62	ATM	p.Q852*	2.33
BA60	ATM	p.S1691R	2.33
	ATM	***Total***	***11.63***
BA33	CDH1	p.G78D	2.33
	CDH1	***Total***	***2.33***
BA37	CDKN2A	p.A68T	2.33
BA14	CDKN2A	p.E61*	2.33
BA17	CDKN2A	p.W110*	2.33
	CDKN2A	***Total***	***6.98***
BA17, BA27, BA3	EGFR	p.L858R	6.97
BA62	EGFR	p.E746_A750del	2.33
BA62	EGFR	p.E746_E749del	2.33
BA55	EGFR	p.S768I	2.33
	EGFR	***Total***	***11.63***
BA25	ERBB2	p.Y772_A775dup	2.33
	ERBB2	***Total***	***2.33***
BA18, BA56	FGFR3	p.F386L	4.65
BA36	FGFR3	p.A719T	2.33
BA25	FGFR3	p.G377D	2.33
	FGFR3	***Total***	***9.30***
BA51	FLT3	p.G583D	2.33
	FLT3	***Total***	***2.33***
BA18, BA72	JAK3	p.V722I	4.65
BA17	JAK3	p.T714A	2.33
	JAK3	***Total***	***6.98***
BA13	KDR	p.E1203*	2.33
BA62	KDR	p.R962H	2.33
BA17	KDR	p.T1350I	2.33
	KDR	***Total***	***6.99***
BA30, BA34, BA40, BA50, BA52, BA55, BA60, BA67	KRAS	p.G12C	18.60

BA15, BA33, BA47, BA66	KRAS	p.G12V	9.30
BA16, BA24, BA53, BA9	KRAS	p.G12D	9.30
BA71, BA72	KRAS	p.G13C	4.65
BA13	KRAS	p.G12A	2.33
BA23	KRAS	p.Q61K	2.33
	KRAS	***Total***	***46.51***
BA71	MLH1	p.R389Q	2.33
BA71	MLH1	p.V384D	2.33
	MLH1	***Total***	***2.33***
BA22	NOTCH1	p.H1601L	2.33
BA13	NOTCH1	p.G2447C	2.33
BA41	NOTCH1	p.R1568G	2.33
	NOTCH1	***Total***	***6.99***
BA62	PDGFRA	p.P653S	2.33
	PDGFRA	***Total***	***2.33***
BA24, BA33, BA36, BA41, BA47, BA51, BA57, BA9	PIK3CA	p.I391M	18.60
BA57	PIK3CA	p.E542K	2.33
BA41	PIK3CA	p.E417Q	2.33
BA54	PIK3CA	p.V344M	2.33
	PIK3CA	***Total***	***20.93***
BA56	PTEN	p.N63Kfs*11	2.33
	PTEN	***Total***	***2.33***
BA26	PTPN11	p.G503V	2.33
	PTPN11	***Total***	***2.33***
BA17, BA22, BA23, BA37, BA53, BA56, BA71	RB1	p.I680T	16.28
BA24, BA30, BA55	RB1	p.T766Nfs*29	6.98
BA71	RB1	p.Q575Tfs*10	2.33
	RB1	***Total***	***23.26***
BA22, BA23, BA56	RET	p.E884V	6.98
BA17	RET	p.E901K	2.33
	RET	***Total***	***9.31***
BA17, BA22, BA23, BA27, BA53, BA56, BA9	SMAD4	p.V354L	16.28
BA17, BA22, BA23, BA53, BA56	SMAD4	p.Q256L	11.63
BA54	SMAD4	p.C363S	2.33
BA40	SMAD4	p.C499R	2.33
BA33	SMAD4	p.R361C	2.33
	SMAD4	***Total***	***23.26***
BA22	SMARCB1	p.I63Nfs*8	2.33
	SMARCB1	***Total***	***2.33***
BA47	SMO	p.A406S	2.33
	SMO	***Total***	***2.33***
BA22, BA66	STK11	p.D194Y	4.65
BA47, BA60	STK11	p.X155_splice	4.65
BA5	STK11	p.L282Sfs*5	2.33
BA32	STK11	p.R40Sfs*6	2.33
	STK11	***Total***	***13.95***

BA24, BA30, BA7	TP53	p.V157F	6.98
BA18, BA26, BA5	TP53	p.A159P	6.98
BA23, BA3	TP53	p.E286V	4.65
BA72, BA9	TP53	p.G266V	4.65
BA12, BA7	TP53	p.R196*	4.65
BA2, BA47	TP53	p.R248L	4.65
BA33, BA72	TP53	p.R158L	4.65
BA3	TP53	p.R273L	2.33
BA22	TP53	p.S96P	2.33
BA40	TP53	p.Y220C	2.33
BA56	TP53	p.A161T	2.33
BA71	TP53	p.C176F	2.33
BA15	TP53	p.C176S	2.33
BA57	TP53	p.E198*	2.33
BA22	TP53	p.E285K	2.33
BA1	TP53	p.G226Wfs*3	2.33
BA56	TP53	p.G302Rfs*4	2.33
BA36	TP53	p.G334V	2.33
BA30	TP53	p.H179Y	2.33
BA67	TP53	p.H193R	2.33
BA54	TP53	p.N247I	2.33
BA63	TP53	p.P82Rfs*41	2.33
BA22	TP53	p.P92S	2.33
BA16	TP53	p.R174W	2.33
BA32	TP53	p.R175H	2.33
BA34	TP53	p.R202L	2.33
BA33	TP53	p.R213*	2.33
BA14	TP53	p.R342*	2.33
BA34	TP53	p.V203L	2.33
BA25	TP53	p.V216L	2.33
BA50	TP53	p.W91Cfs*30	2.33
	TP53	***Total***	***72.09***

[1] Total % Frequency is defined as total number of cases with one or more variant in a particular gene divided total number of cases.

Supplemental Table 7: Variants found in *MET*-altered LUAD BM cases (*N* = 31).

Case Number	Hugo_Symbol	HGVSp_Short	Frequency (%)
**BA39**, **BA48**	ABL1	p.G278V	6.45
**BA38**	ABL1	p.C349YT	3.23
**BA35**	ABL1	p.S429T	3.23
	ABL1	***Total***	***12.90***
**BA39**, **BA42**, **BA48**	ALK	p.G1184E	9.68
**BA39**, **BA42**	ALK	p.K1173I	6.45
	ALK	***Total***	***9.68***[1]
**BA38**, **BA39**, **BA42**, **BA48**, **BA59**, **BA73**	APC	p.P1433L	19.35
**BA39**, **BA48**	APC	p.A888T	6.45
**BA61**	APC	p.E1317Q	3.23
**BA48**	APC	p.Q1095R	3.23
	APC	***Total***	***22.58***
**BA42**, **BA58**	ATM	p.F858L	6.45
**BA42**	ATM	p.L2885P	3.23
	ATM	***Total***	***6.45***
**BA45**	CDKN2A	p.D108V	3.23
**BA31**	CDKN2A	p.E69Qfs*49	3.23
**BA4**	CDKN2A	p.H83Y	3.23
**BA46**	CDKN2A	p.R58*	3.23
**BA48**	CDKN2A	p.R80*	3.23
	CDKN2A	***Total***	***16.13***
**BA38**, **BA39**, **BA42**, **BA48**	EGFR	p.T783A	12.9
**BA38**, **BA42**, **BA48**	EGFR	p.A822T	9.68
**BA38**	EGFR	p.E746_A750del	3.23
**BA38**	EGFR	p.G810S	3.23
	EGFR	***Total***	***12.91***
**BA39**, **BA48**	FBXW7	p.T482A	6.45
	FBXW7	***Total***	***6.45***
**BA39**, **BA42, BA48**	FGFR3	p.N646D	9.68
	FGFR3	***Total***	***9.68***
**BA39**, **BA48**	FLT3	p.K602R	6.45
**BA39**	FLT3	p.S684P	3.23
	FLT3	***Total***	***6.45***
**BA48**	GNA11	p.G208C	3.23
	GNA11	***Total***	***3.23***
**BA39**, **BA48**	IDH1	p.L120P	6.45
**BA39**, **BA42**	IDH1	p.P118L	6.45
	IDH1	***Total***	***9.68***
**BA48**	IDH2	p.K155N	3.23
**BA38**	IDH2	p.W164*	3.23
	IDH2	***Total***	***6.46***
**BA35**	JAK3	p.V722I	3.23
	JAK3	***Total***	***3.23***

**BA39**	KDR	p.G1348E	3.23
	KDR	***Total***	***3.23***
**BA11**, **BA35**, **BA46, BA68**	KRAS	p.G12C	12.90
**BA48**, **BA49**, **BA58**	KRAS	p.Q61H	9.68
**BA4**, **BA8**	KRAS	p.G12A	6.45
**BA6**, **BA74**	KRAS	p.G12V	6.45
**BA19**, **BA20**	KRAS	p.G13D	6.45
**BA64**	KRAS	p.G12D	3.23
**BA65**	KRAS	p.Q61L	3.23
**BA73**	KRAS	p.Q61R	3.23
	KRAS	***Total***	***51.61***
**BA11**, **BA20**, **BA21**, **BA4**, **BA43**, **BA44**	MET	p.N375S	19.35
**BA6**, **BA38**, **BA58**, **BA59**, **BA70**, **BA74**	MET	p.T1010I	19.35
**BA39**, **BA42**, **BA48**	MET	p.A179T	6.68
**BA29**	MET	p.H1112Y	3.23
**BA38**	MET	p.H855Y	3.23
	MET	***Total***	***51.61***
**BA39**	MLH1	p.Q391H	3.23
	MLH1	***Total***	***3.23***
**BA74**	NOTCH1	p.E1583D	3.23
**BA42**	NOTCH1	p.H1601L	3.23
**BA74**	NOTCH1	p.M1580L	3.23
**BA74**	NOTCH1	p.R1598H	3.23
**BA74**	NOTCH1	p.R1598H	3.23
	NOTCH1	***Total***	***6.45***
**BA39**	NRAS	p.G12V	3.23
		Total	***3.23***
**BA19**, **BA20**, **BA38**, **BA44**, **BA49**, **BA65**, **BA73**	PIK3CA	p.I391M	22.58
**BA38**	PIK3CA	p.E542K	3.23
**BA74**	PIK3CA	p.M1040I	3.23
	PIK3CA	**Total**	***25.80***
**BA38**	PTEN	p.K267Rfs*9	3.23
	PTEN	***Total***	***3.23***
**BA38**, **BA39**, **BA42**, **BA48**, **BA73**	RB1	p.I680T	16.13
**BA39**, **BA42**, **BA48**	RB1	p.L670P	9.68
	RB1	***Total***	***16.13***
**BA39**, **BA42**, **BA48, BA59**, **BA73**	RET	p.E884V	16.13
**BA39**	RET	p.D892Y	3.23
**BA48**	RET	p.M918V	3.23
	RET	***Total***	***16.13***
**BA38**, **BA39**, **BA42**, **BA48**, **BA73**	SMAD4	p.V354L	16.13
**BA39**, **BA42**, **BA48**, **BA59**	SMAD4	p.Q256L	12.90
**BA48**	SMAD4	p.I347V	3.23
**BA49**	SMAD4	p.R189C	3.23
	SMAD4	***Total***	***22.58***
**BA48**	SMARCB1	p.E59*	3.23

	SMARCB1	***Total***	***3.23***
**BA58**	STK11	p.E199*	3.23
**BA4**	STK11	p.E57Kfs*7	3.23
**BA10**	STK11	p.I274Sfs*13	3.23
**BA6**	STK11	p.P281Rfs*6	3.23
	STK11	***Total***	***12.91***
**BA38**, **BA42**, **BA70**	TP53	p.R273L	9.68
**BA10**, **BA39**	TP53	p.V157F	6.45
**BA21**, **BA61**	TP53	p.R158L	6.45
**BA58**, **BA65**	TP53	p.R248Q	6.45
**BA39**, **BA48**	TP53	p.V97A	6.45
**BA43**, **BA74**	TP53	p.X307_splice	6.45
**BA38**	TP53	p.R282W	3.23
**BA45**	TP53	p.C242F	3.23
**BA35**	TP53	p.C275Lfs*31	3.23
**BA29**	TP53	p.E294*	3.23
**BA48**	TP53	p.E298*	3.23
**BA49**	TP53	p.G154V	3.23
**BA46**	TP53	p.H193P	3.23
**BA64**	TP53	p.K351*	3.23
**BA68**	TP53	p.P151H	3.23
**BA68**	TP53	p.P152Afs*28	3.23
**BA8**	TP53	p.Q136E	3.23
**BA69**	TP53	p.Q167*	3.23
**BA73**	TP53	p.R110L	3.23
**BA28**	TP53	p.R333Lfs*12	3.23
**BA44**	TP53	p.S215I	3.23
**BA42**	TP53	p.S96P	3.23
**BA74**	TP53	p.Y220C	3.23
	TP53	***Total***	***77.42***
**BA38**, **BA39**, **BA42**, **BA48**	VHL	p.V142G	12.90
**BA74**	VHL	p.V87L	3.23
	VHL	***Total***	***16.13***

Blue = *MET*-mutant cases; **Red** = *MET*-amplified cases; **Purple** = *MET*-mutant and amplified cases

[1] Total % Frequency is defined as total number of cases with one or more variant in a particular gene divided total number of cases.

Supplemental References

1. Kumar V, et al. TWIST1 is a critical downstream target of the HGF/MET pathway and is required for MET driven acquired resistance in oncogene driven lung cancer. *Oncogene.* 2024;43(19):1431-44.
2. Kris MG, et al. Using multiplexed assays of oncogenic drivers in lung cancers to select targeted drugs. *JAMA : the Journal of the American Medical Association.* 2014;311(19):1998-2006.
3. Stabile LP, et al. Syngeneic tobacco carcinogen-induced mouse lung adenocarcinoma model exhibits PD-L1 expression and high tumor mutational burden. *JCI insight.* 2020.
4. Finotello F, et al. Molecular and pharmacological modulators of the tumor immune contexture revealed by deconvolution of RNA-seq data. *Genome Med.* 2019;11(1):34.
5. Mayakonda A, et al. Maftools: efficient and comprehensive analysis of somatic variants in cancer. *Genome research.* 2018;28(11):1747-56.
6. Bray NL, et al. Near-optimal probabilistic RNA-seq quantification. *Nat Biotechnol.* 2016;34(5):525-7.
7. Soneson C, et al. Differential analyses for RNA-seq: transcript-level estimates improve gene-level inferences. *F1000Res.* 2015;4:1521.
8. Robinson MD, and Oshlack A. A scaling normalization method for differential expression analysis of RNA-seq data. *Genome Biology.* 2010;11(3):R25.
9. Law CW, et al. voom: precision weights unlock linear model analysis tools for RNA-seq read counts. *Genome Biology.* 2014;15(2):R29.
10. Alhamdoosh M, et al. Combining multiple tools outperforms individual methods in gene set enrichment analyses. *Bioinformatics.* 2017;33(3):414-24.
11. Korotkevich G, et al. Fast gene set enrichment analysis. *bioRxiv.* 2021:060012.
12. Liberzon A, et al. The Molecular Signatures Database (MSigDB) hallmark gene set collection. *Cell Syst.* 2015;1(6):417-25.
13. Aran D, et al. xCell: digitally portraying the tissue cellular heterogeneity landscape. *Genome Biology.* 2017;18(1):220.
